# Reliable two-stage location problem for mitigating flood impact on sustainable agricultural production and waste management

**DOI:** 10.1007/s10479-026-07293-9

**Published:** 2026-06-12

**Authors:** Trung Hieu Tran, Thu Ba T. Nguyen

**Affiliations:** 1 Loughborough Business School, Loughborough University, Loughborough LE11 3TU, Loughborough, UK; 2 Center for Transportation & Logistics, Massachusetts Institute of Technology, Cambridge, MA 02142 USA; 3 Graduate School of Business, University of Bedfordshire, Luton LU1 3JU, UK

**Keywords:** Location, Sustainable waste management, Disruption risk, Probability chains, Two-stage solution method, Math-heuristic algorithm

## Abstract

Sustainable agricultural production and waste management, i.e., stopping burning agricultural waste at fields and utilising the agricultural waste to produce biomass energy for improving the quality of environment, and enhancing the development of regional economy and society, have recently posed challenges due to impact of climate change (e.g., disruption risk by flooding on agricultural waste collection and transport network from fields to treatment facilities). In this paper, we develop a mixed-integer nonlinear programming model for the reliable agricultural waste collection, transport and treatment network design under impact of flooding that aims to minimise the impact of disruption risk on transport road network and locations of storages and facilities. The model can support rural planners to optimally locate storages (for collecting and transporting agricultural waste from fields to storages) and treatment facilities (for collecting and transporting agricultural waste from storages to treatment facilities). In the novel reliable two-stage location problem, the overall objective is to minimise total expected “disruption risk” cost. A solution technique based on “probability chains” is developed to transform the mixed-integer nonlinear programming model into a quadratic programming model that can be solved by advanced optimisation solvers. Based on the property of problem decomposition, we propose a two-stage solution method to seek quickly the optimal solutions of the reliable two-stage location problem. In addition, we develop an efficient math-heuristic algorithm for solving the large-sized instances within a reasonable computation time. The efficiency of the proposed solution methods is evaluated on the case studies in the Vietnam Mekong Delta.

## Introduction

Sustainable agricultural production and waste management has been considered as an innovative solution to support the developing countries, whose economic growth contribution mainly depends on the agricultural development, to mitigate the impact of climate change on their agricultural sectors. Recently, the innovative practice has been implemented and brought huge benefits for farmers at Quang Tri, Vietnam (Tran et al., 2024). However, a sustainable agricultural production and waste management model under disruption risk caused by flooding has not been investigated. Hence, it is challenging to apply and implement the model at a region in which there exist high disruption risks on road network and sites.

**Fig. 1:**
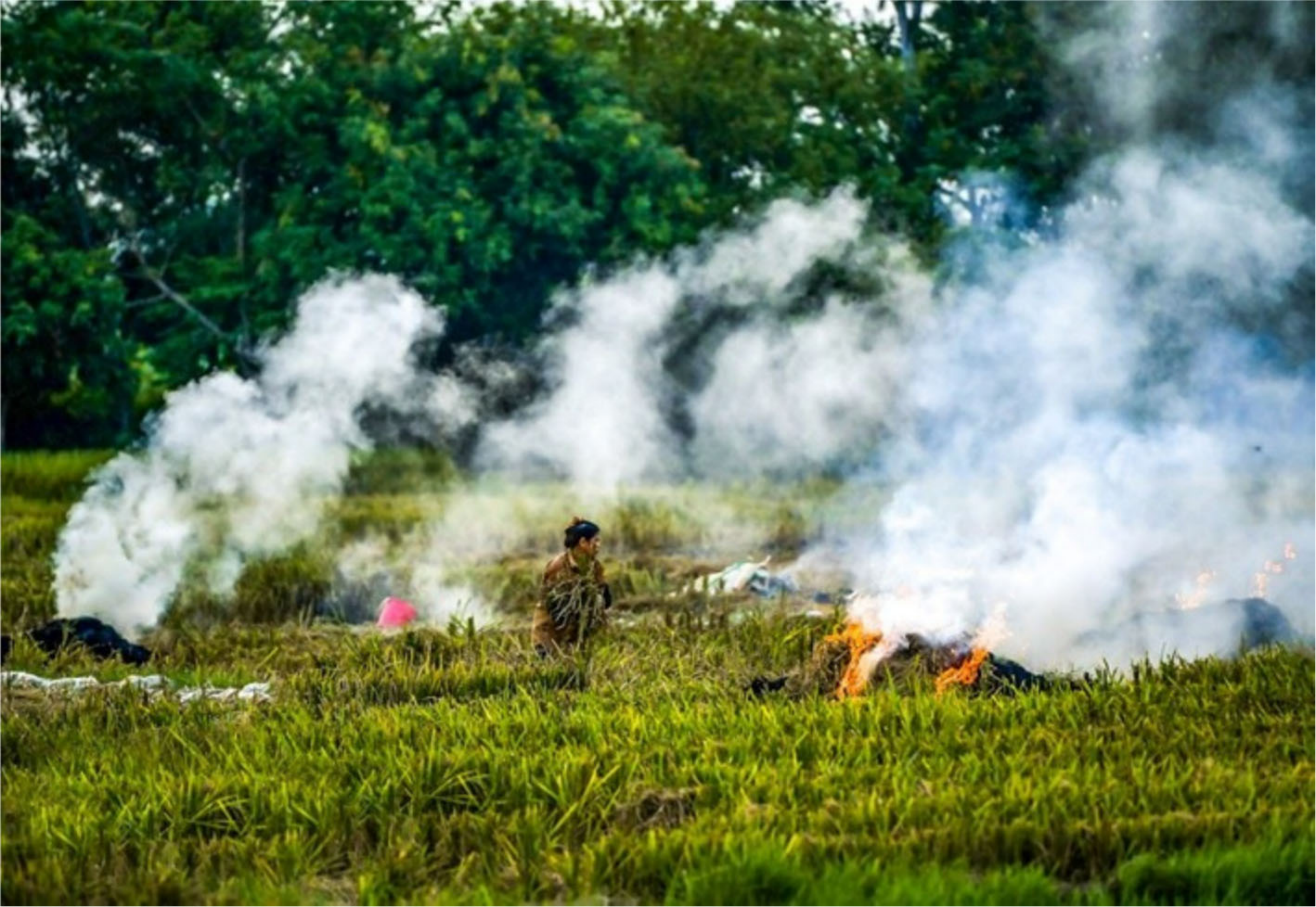
Agricultural waste burning, a common practice at the Vietnam Mekong Delta. Source: https://amdi.vn/en/news--events/amdi-conducts-research-on-sustainable-agricultural-waste-management-at-the-mekong-delta.html.

The Mekong Delta plays an important role in Vietnam’s agricultural development. Although its area is about 12% of Vietnam area, it contributes more than 90% of Vietnam’s exported rice quantity. As a result, the annual quantity of rice straw is 26.2 million tons (i.e., 42% of Vietnam’s rice straw), of which 20.9 million tons are burned directly on the fields, which contributes approximately 17.95 million tons of CO2, 485.58 thousand tons of CO and 10.38 thousand tons of NOx into the atmosphere. Most farmers intend to continue the bad practice of burning rice straw at fields in the coming years (see Figure 1), which will cause increasingly the air, land and water pollution in the Vietnam Mekong Delta.

**Fig. 2:**
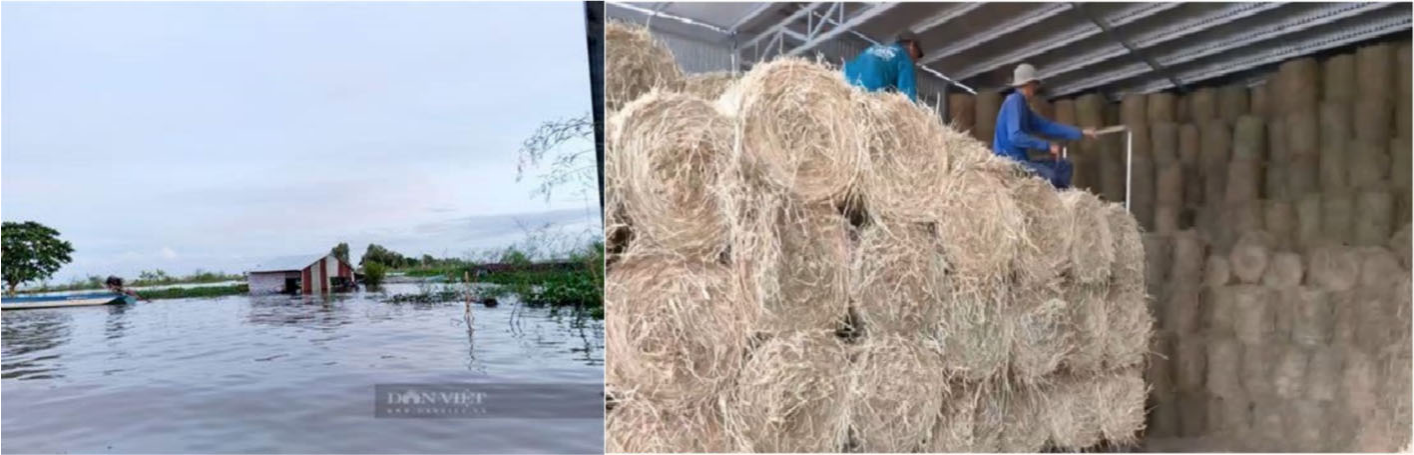
An image of the impact of flooding on agricultural waste collection and transport network. (Source: https://danviet.vn/an-giang-canh-bao-dinh-trieu-cuong-gay-ngap-lut-tu-ngay-26-28-10-20221024182633206-d1052023.html)

Flooding in the Vietnam Mekong Delta frequently leads to road submergence, reduced accessibility, and temporary isolation of storage and facility sites (see Figure 2). These disruption mechanisms directly influence how agricultural waste can be collected and transported during the post-harvesting period. To represent these realities in an analytically tractable way, we translate flood impacts into failure probabilities on both road links and facility sites, capturing the likelihood that a route becomes impassable or a site becomes inaccessible. This interpretation leads to compound probability expressions, such as sequential link failures or simultaneous road and site disruptions, which introduce non-linearities into the mathematical model. Hence, there is an urgent need of innovative model development for dealing with the challenge.

To address the challenge, we propose a novel reliable location model for rural planners to design a reliable agricultural waste collection, transport and treatment network under impact of climate change (e.g., disruption risk by flooding) towards sustainable agricultural production and waste management in the Vietnam Mekong Delta. The model can determine the optimal storage locations for collecting agricultural waste from fields to storages and the optimal facility locations for collecting agricultural waste from storages to treatment facilities. This is a reliable two-stage location problem (namely, RTLP), an extension of the reliable facility location problem (O’Hanley et al., 2013), where we take account into the impact of flooding risk on agricultural waste transport network and locations of storages and facilities (i.e., failure probabilities on roads and sites). The overall objective of the RTLP is to minimise total expected “disruption risk” cost of locating waste storages and facilities, and collecting agricultural waste from fields to storages as well as from storages to facilities. A mixed-integer nonlinear programming (MINLP) model for this problem is formulated. The MINLP model is transformed into a quadratic programming model by a novel two-stage “probability chains” technique (O’Hanley et al., 2013; Tran et al., 2017) so that it can be solved using an advanced optimisation solver. A two-stage solution method is proposed to find quickly the optimal solutions of the RTLP. In addition, we construct a math-heuristic algorithm, combing solving the mathematical programming model and the tabu search, for solving the large-sized instances within a reasonable computation time. The mathematical models and solution methods are evaluated in the case studies of reliable agricultural waste collection, transport and treatment network design in the Vietnam Mekong Delta.

The remaining of this paper is organized as follows. A literature review is presented in Section 2. Section 3 describes mathematical programming models and solution methods. Section 4 is the case studies and the experimental results of solving the case studies by the proposed solution methods. Finally, Section 5 provides a conclusion of long-term impact, limitation and future work.

## Literature review

Li et al. (2025) have recently done a literature review of state-of-the-art of sustainable waste collection and vehicle routing problem (WCVRP). The literature review emphasize that there are a few of research works on sustainable agricultural waste management though it has a high importance.

For example, Wang et al. (2023) integrated the concept of green cycle development and the relevant theories into modern system engineering to optimise the problem of agricultural production waste recycling network, as know as traditional location routing problem (LRP). Their model can select the optimal facility sites and plan the optimal vehicle routes for the minimisation of facility construction and operational costs. A genetic algorithm is developed to solve efficiently the large-sized instances. The feasibility and efficiency of the model and algorithm are validated through computational experiments. Their study emphasize that agricultural waste resource utilisation can contribute the development of an ecological agriculture cycle. Tran et al. (2024) then provided a summary of the various applications of traditional LRP in practice. The authors also investigated a novel location-assignment-routing problem (LARP) for an agricultural waste collection and transport network design (without uncertainty) to collect completely and efficiently agricultural waste from fields, in the purpose of environmental quality improvement and economic development. In the novel LARP, the overall objective is to minimise total cost of locating storages, collecting waste from fields and planning vehicle routes for transporting waste from storages to the facility. A mathematical programming model is proposed to solve optimally the problem, and a parallel water flow algorithm is developed to solve efficiently the large-sized instances within reasonable computational time. The efficiency of the proposed model and algorithm is validated and evaluated on the real case study in Trieu Phong district, Quang Tri province, Vietnam, as well as a set of randomly generated large-sized instances. The computational results show that their solution approach outperforms the general optimisation solver and the tabu search. However, both research works have not taken account into the impact of climate change (e.g., disruption risk due to flooding) on the agricultural waste collection and transport network design.

For studying impact of uncertainty on other waste management problem, Spinelli et al. (2025) have recently proposed a multi-stage stochastic optimisation model for the collection of recyclable municipal waste. This is an inventory routing problem that aims to maximize the total expected profit of the waste collection company by the selection of the bins to be visited and the corresponding routing plan in a predefined time horizon. The authors model stochasticity in waste accumulation for each route through scenario trees generated via conditional density estimation and dynamic stochastic approximation techniques, and provide a rigorous worst-case analysis on performance. Computational experiments are carried out on large-sized instances, based on the real-world data provided by a large Portuguese waste collection company. The impact of stochasticity on waste generation is examined through stochastic measures, showing the importance of adopting a stochastic model over a deterministic formulation when addressing a waste collection problem. The results also demonstrate the efficiency of the proposed heuristic for seeking cost-effective solutions in short computational time. However, this optimisation model cannot be extended and applied for the agricultural waste management problem under uncertainty since there are many different characteristics between both agricultural and municipal wastes. In addition, their model has not considered the decision variables of storage and facility locations in the stochastic optimisation problem, and the impact of climate change (e.g.., disruption risk by flooding) on waste collection and transport network design.

Furthermore, these previous studies have mainly focused on node failures because most the reliable facility location models conceptualize disruptions as site specific events that disable facilities or depots. For example, the “probability chain” formulation by O’Hanley et al. (2013) modeled reliability through failure probabilities at facility sites, without incorporating link reliability. Similarly, the recent agricultural and municipal waste management models, e.g., Wang et al. (2023), Tran et al. (2024) and Spinelli et al. (2025), treated disruption risk either as uncertainty in waste generation or as site based reliability, but do not include link failures in their mathematical formulations. The main reason that link failures have been neglected is due to the methodological issue, i.e., integrating link-level disruption probabilities together with node failures significantly increases modeling and computational complexity. Capturing simultaneously both node and link failures requires: (i) re-defining the probability structure governing service feasibility, (ii) re-engineering the assignment logic so that both site and route disruptions are considered jointly, and (iii) introducing additional non-linear compound probability terms that most existing linearisation or stochastic optimisation frameworks cannot handle efficiently.

Recent research on resilient facility location and network design has increasingly emphasized the integration of disruption risk, uncertainty, and climate-related hazards into strategic planning decisions. A growing body of work employs stochastic, robust, or scenario-based optimisation frameworks to model the impact of disruptions on infrastructure and logistics networks, demonstrating that explicitly accounting for failures can substantially alter optimal network configurations and improve system resilience. These studies span applications such as supply chain design, humanitarian logistics, and critical infrastructure protection, highlighting the importance of proactive resilience-oriented planning (Eberhardt et al., 2026; Hu et al., 2026; Sabouhi et al., 2025).

Despite these advances, most existing models are formulated for single-echelon network structures and primarily represent disruptions through node-level failures, while transport link failures are often treated implicitly or omitted. Moreover, many approaches rely on scenario enumeration or stochastic programming formulations that can become computationally burdensome for large-scale instances. In contrast, the present study considers a two-echelon agricultural waste collection system and explicitly incorporates both site and transport-link failures induced by flooding. Furthermore, we adopt an exact probability-chain reformulation that preserves the reliability structure of the problem while enabling tractable mixed-integer optimisation. This allows the proposed model to extend existing reliability-oriented location frameworks to multi-echelon, flood-prone waste management networks.

Table 1 provides a summary of relevant research works on waste collection routing problem under uncertainties, and resilient facility location and network design. To the best of our knowledge, no prior agricultural waste or facility location study has simultaneously addressed both types of failures within a unified optimisation framework. Hence, our main contributions include:

- Formulating a MINLP model for solving the RTLP in the sustainable agricultural production and waste management under impact of flooding.
- Developing a two-stage “probability chain” technique to transform the MINLP model into a quadratic programming model that can be solved by an advanced optimisation solver.
- Proposing a two-stage solution method for seeking quickly the optimal solutions of the RTLP.
- Constructing an efficient math-heuristic algorithm for solving the large-sized instances within a reasonable computation time.
- Implementing the models and solution methods for the case studies in the Vietnam Mekong Delta.

**Table 1:** A summary of relevant research works on waste collection routing problem under uncertainties.

Reference	Application	Uncertainty	Problem	Solution method
Nuortio et al. (2006)	Solid waste	Bins’ filling rate,	Stochastic periodic VRP	MILP, Guided variable
		Travel time	with time windows	neighborhood thresholding
Kuo et al. (2012)	Solid waste	Bins’ filling rate	Capacitated VRP	ILP, PSO and GA
			with fuzzy demand	
Mes et al. (2014)	Solid waste	Bins’ filling rate,	Stochastic dynamic IRP	MILP, Problem-based
		Travel time		heuristic algorithm
Nolz et al. (2014)	Medical product	Bins’ filling rate	Stochastic IRP	MILP, ALNS
Elbek and Wohlk (2016)	Solid waste	Bins’ filling rate	Multi-product	MILP, VNS
			multi-period IRP	and rolling horizon
Gruler et al. (2017)	Not specified	Bins’ filling rate	Multi-depot VRP	MILP, Iterated local search
			with stochastic demand	and Monte Carlo simulation
Soysal et al. (2018)	Food waste	Customer demand	Chance-constrained IRP	MILP, Exact Solver
Markov et al. (2020)	Solid waste	Bins’ filling rate	Stochastic IRP	MINLP, ALNS, SA
				and rolling horizon
Aliahmadi et al. (2021)	Solid waste	Bins’ filling rate	Capacitated VRP	MILP, Augmented
			with hard time windows	ε-constraint NSGA II
Zhou et al. (2021)	Textile	Pick-up level	Chance-constrained VRP	MILP, GA
			with pickup and delivery	
Gholizadeh et al. (2022)	Polystyrene	Demand cost	Robust IRP	MINLP, GA, SA
				and cross entropy
Sadrabadi et al. (2024)	Recyclable	Cost, Amount of waste,	Stochastic IRP	MINLP, NSGA II
	hazardous waste	Population risk		
Spinelli et al. (2025)	Recyclable	Bins’ filling level	Multi-stage stochastic IRP	MILP, Rolling horizon
	municipal waste	over multiple periods		
Zhang et al. (2025)	Agricultural waste	Behaviors of farmers and	Agricultural waste recovery	Game theory approach
		manufacturing enterprises	and organic fertilizer	
			production supply chain	
Sabouhi et al. (2025)	Supply chain	Facility disruptions,	Resilient supply chain	Stochastic-robust optimisation,
	network design	operational risks	network design	Benders decomposition
Eberhardt et al. (2026)	Disaster relief	Warehouse availability, route	Cooperative covering	Scenario-based stochastic
	stockpiling	failures, demand uncertainty	location problem	programming
Hu et al. (2026)	Supply chain	Cascading node failures	Network-level resilience	Simulation-based adaptive
	resilience		and recovery planning	modelling
This paper	Agricultural waste	Disruption risk	Reliable two-stage location problem	MINLP, MIQP, Probability chains,
		on road network and sites		Two-stage solution method,
				math-heuristic algorithm

Note that VRP = Vehicle Routing Problem, IRP = Inventory Routing Problem, LARP = Location Assignment Routing Problem, ILP = Integer Linear Programming, MILP = Mixed-Integer Linear Programming, MINLP = Mixed-Integer Nonlinear Programming, PSO = Particle Swarm optimisation, SA = Simulated Annealing, GA = Genetic Algorithm, VNS = Variable Neighborhood Search, ALNS = Adaptive Large Neighborhood Search, NSGA = Non-dominated Sorting Genetic Algorithm

## Sustainable agricultural production and waste management under disruption risk

This section introduces the reliable two-stage location problem (RTLP) and presents the modelling framework and solution approaches adopted in this study. We first describe the problem context and key assumptions, then introduce the mathematical formulation and probability-chain reformulation. Finally, we outline the exact two-stage solution method and the math-heuristic algorithm developed to address scalability for large-scale instances.

### Reliable two-stage location problem under flood disruption

**Fig. 3:**
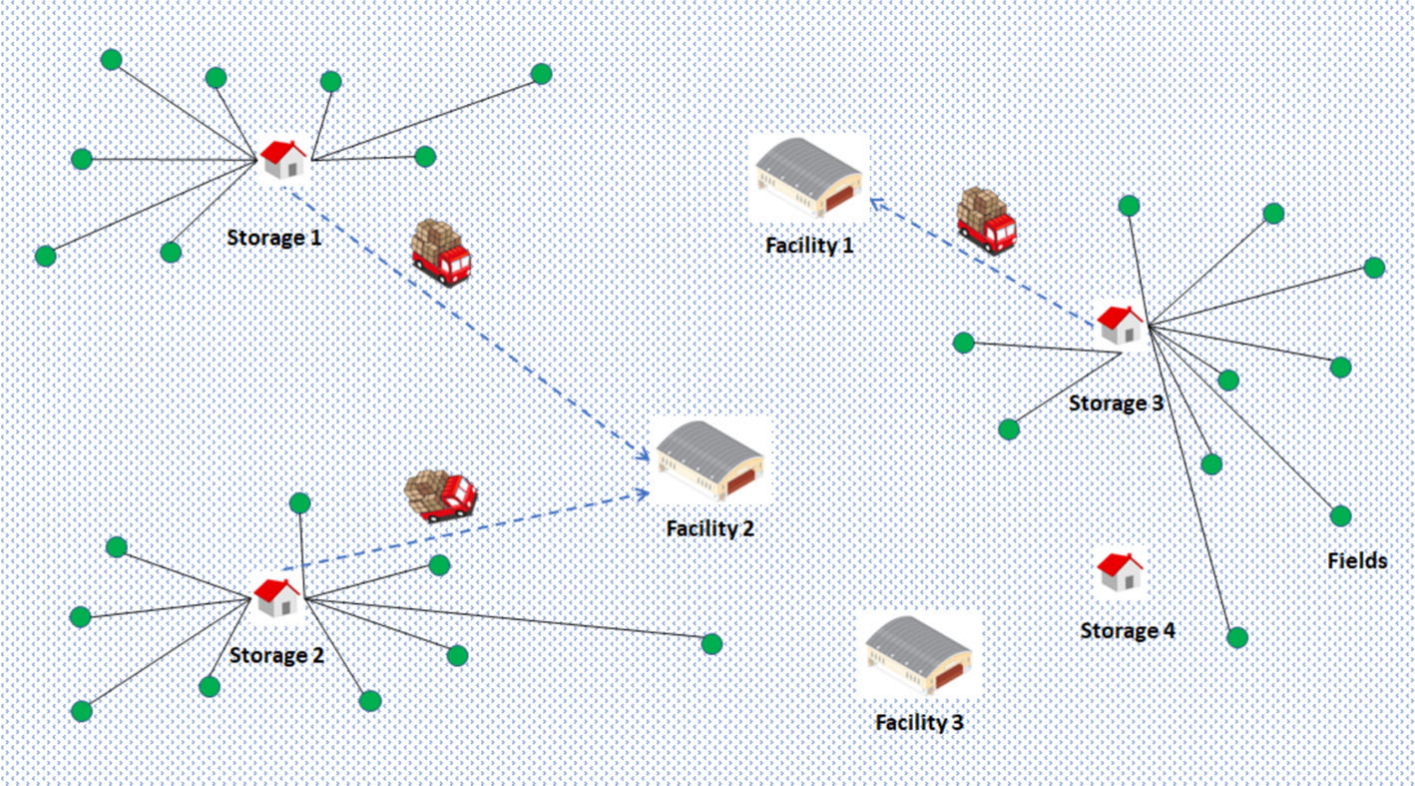
An example of the RTLP.

Figure 3 is an example of the RTLP for the design of sustainable agricultural waste collection and transport network under impact of flooding. In the problem, a set of waste storages are located to collect and temporarily store agricultural waste from fields before being transported to treatment/ production facilities. Each field is assigned to only one storage. The assignment of field and storage depends on the distance between field and storage, the disruption risks of transport road and storage site, and the demand of field. In addition, each storage is assigned to only one facility. The assignment of storage and facility depends on the distance between storage and facility, the disruption risks of transport road, storage and facility sites, and the demand of storage. Since the impact of flooding (e.g., disruption risk) on the collection and transport network and the sites of storages and facilities are addressed in the problem, we simultaneously consider seeking the optimal and reliable sets of storage and facility locations for the minimisation of total expected “disruption risk” cost.

**Fig. 4:**
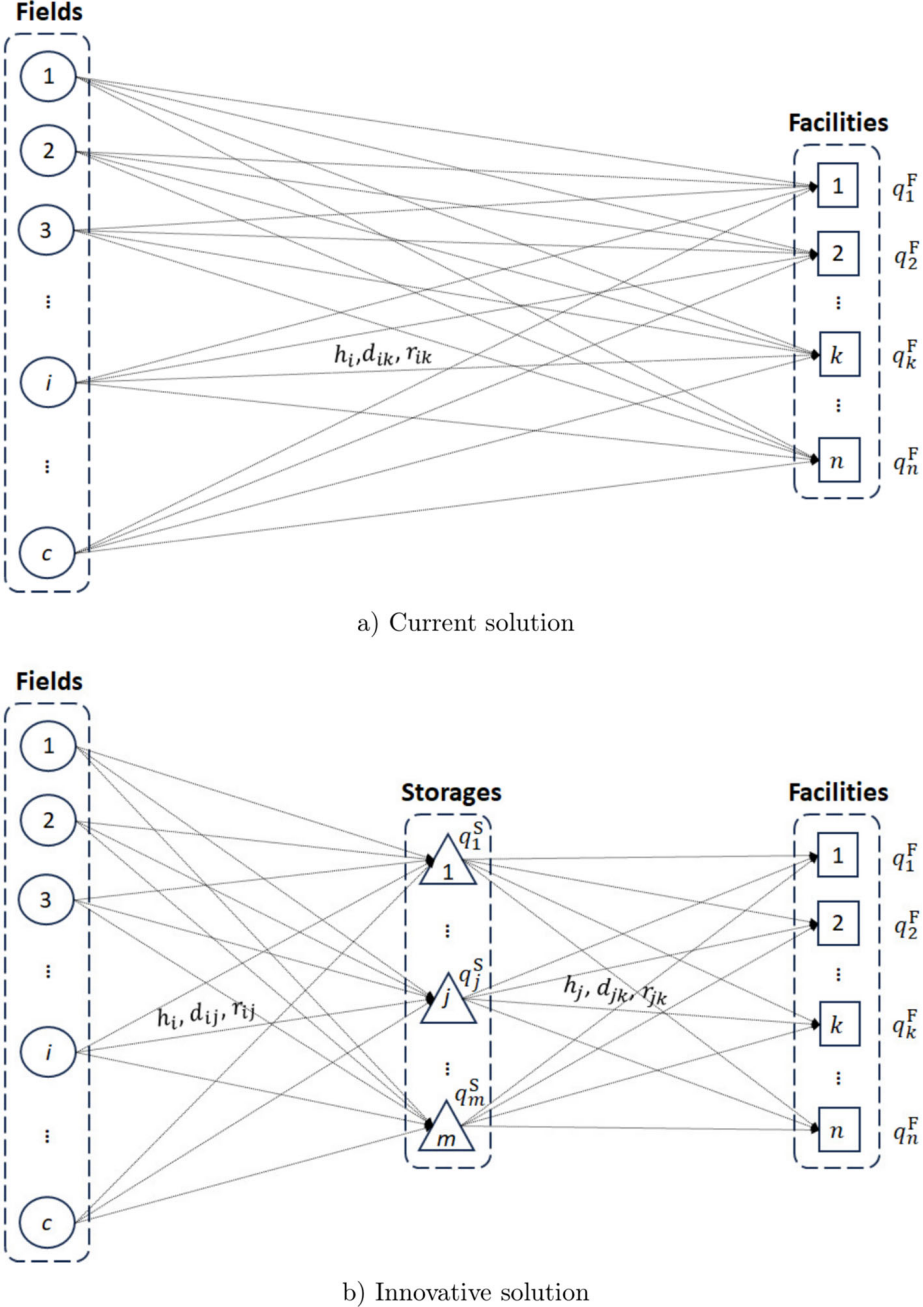
A schematic illustration of the innovative solution for the RTLP.

Figure 4 is a schematic illustration for the RTLP, in which Figure 4a is the current solution implementing in the Vietnam Mekong Delta, and Figure 4b is the innovative solution proposed (i.e., temporary waste storages are located to collect agricultural waste from fields). Note that each field is assigned to only one storage, and each storage is assigned to only one facility.

In this problem, note that “two-stage” refers to the two-echelon architecture of the sustainable agricultural waste collection and transport network (Stage 1: field → storage, and Stage 2: storage→ facility). This problem, however, is still a single-level mathematical programming model that jointly determines storage and facility locations and induced assignments while accounting for compounded link and site failures. The terminology of Stage 1 and Stage 2 denotes an algorithmic decomposition to enhance tractability rather than a formal Stackelberg bi-level program.

**Table 2:** The list of notations.

Notation	Description
Sets and indexes:	
*I*	Set of agricultural waste fields (indexed by *i*)
*J*	Set of storages (indexed by *j*)
*K*	Set of facilities (indexed by *k*)
$i_{s},i_{\ell }$	Denote the *s*th, ℓth closest storage to field *i*
$j_{t},j_{\ell }$	Denote the *t*th, ℓth closest facility to storage *j*
$\overline{i}_{j}$	Denote the closest rank (th) of storage *j* to field *i*
Parameters:	
*c*	Number of fields (i.e., c=|I|)
*m*	Number of storages (i.e., m=|J|)
*n*	Number of facilities (i.e., n=|K|)
$h_{i},h_{j}$	Amount of agricultural waste collected at field *i*, storage *j*
$d_{ij},d_{jk}$	Cost (or travel distance) between field *i* and storage *j*, between storage *j* and facility *k*
$r_{ij},r_{jk}$	Risk of disruption (due to flooding) on link (*i*, *j*), link (*j*, *k*)
$q_{j}^{\textrm{S}},q_{k}^{\textrm{F}}$	Failure probability at storage *j*, facility *k*
$\overline{q}_{j}^{\textrm{S}},\overline{q}_{k}^{\textrm{F}}$	Successful probability at storage *j*, facility *k* (i.e., $\overline{q}_{j}^{\textrm{S}}=1-q_{j}^{\textrm{S}}$, $\overline{q}_{k}^{\textrm{F}}=1-q_{k}^{\textrm{F}}$)
$\phi _{1},\phi _{2}$	Penalty cost for all storages failed, all facilities failed
$p_{1},p_{2}$	Number of opened storages, number of opened facilities
Decision variables:	
$$X_{j}={\left\{ \begin{array}{ll} \begin{array}{cc} 1 & \text {if a storage is located at site}{ j\in J}\\ 0 & \text {otherwise} \end{array}\end{array}\right. }$$	
$$Y_{k}={\left\{ \begin{array}{ll} \begin{array}{cc} 1 & \text {if a facility is located at site}{ k\in K}\\ 0 & \text {otherwise} \end{array}\end{array}\right. }$$	

**Independence assumption.**

In this study, link (road) failures and site (storage or facility) failures are modeled as independent conditional on flood exposure. This assumption constitutes a deliberate mathematical simplification adopted to preserve the tractability of the probability-chain formulation and the resulting optimisation models. Although a physical flood event may induce spatially correlated disruptions (i.e., the simultaneous failure of a road segment and an adjacent facility) explicitly modeling such correlations would substantially complicate the probability structure and eliminate the decomposability and linearisation properties exploited in this work. By conditioning on a common flood-exposure layer and assuming residual independence at the asset level, the model thus captures the primary effect of flooding while maintaining computational feasibility for large-scale instances. This assumption enables exact probability-chain representations and scalable solution methods.

### Mathematical notations

Table 2 presents the mathematical notations in the formulation of RTLP. The notations include sets and indexes, parameters, and decision variables that are used in both the MINLP and MILP models.

### Mixed-integer nonlinear programming model

As mentioned above, a single-level mathematical programming model is formulated to solve the RTLP. This model aims to seek the most reliable storage and facility locations, and the optimal assignments of fields and storages, as well as storages and facilities, such that total expected “disruption risk” cost is minimised.

[RTLP-NP]:

1 $$\begin{aligned} &  \min \quad {\displaystyle \sum _{i\in I}}h_{i}\left( {\displaystyle \sum _{s\le m}}d_{ii_{s}}r_{ii_{s}}\prod _{\ell <s}\left( 1-\overline{q}_{i_{\ell }}^{\textrm{S}}X_{i_{\ell }}\right) \overline{q}_{i_{s}}^{\textrm{S}}X_{i_{s}}+\phi _{1}{\displaystyle \prod _{\ell \le m}}\left( 1-\overline{q}_{i_{\ell }}^{\textrm{S}}X_{i_{\ell }}\right) \right) \end{aligned}$$

2 $$\begin{aligned} &  \quad +{\displaystyle \sum _{j\in J}}h_{j}\left( {\displaystyle \sum _{t\le n}}d_{jj_{t}}r_{jj_{t}}\prod _{\ell<t}\left( 1-\overline{q}_{j_{\ell }}^{\textrm{F}}Y_{j_{\ell }}\right) \overline{q}_{j_{t}}^{\textrm{F}}Y_{j_{t}}+\phi _{2}{\displaystyle \prod _{\ell \le n}}\left( 1-\overline{q}_{j_{\ell }}^{\textrm{F}}Y_{j_{\ell }}\right) \right) \nonumber \\ &  \text {s.t.:}{\ h_{j}=\sum _{i\in I}h_{i}\left( \prod _{\ell <\overline{i}_{j}}\left( 1-\overline{q}_{i_{\ell }}^{\textrm{S}}X_{i_{\ell }}\right) \overline{q}_{j}^{\textrm{S}}X_{j}\right) ,\ \forall j\in J,} \end{aligned}$$

3 $$\begin{aligned} &  \quad \sum _{j\in J}X_{j}=p_{1}, \end{aligned}$$

4 $$\begin{aligned} &  \quad \sum _{k\in K}Y_{k}=p_{2}, \end{aligned}$$

5 $$\begin{aligned} &  \quad X_{j}\in \{0,1\},\ \forall j\in J, \end{aligned}$$

6 $$\begin{aligned} &  \quad Y_{k}\in \{0,1\},\ \forall k\in K. \end{aligned}$$

In this model, the objective function (1) aims to minimise total expected “disruption risk” cost that includes the expected “disruption risk” cost of storage location:

$${\displaystyle \sum _{i\in I}}h_{i}\left( {\displaystyle \sum _{s\le m}}d_{ii_{s}}r_{ii_{s}}\prod _{\ell <s}\left( 1-\overline{q}_{i_{\ell }}^{\textrm{S}}X_{i_{\ell }}\right) \overline{q}_{i_{s}}^{\textrm{S}}X_{i_{s}}+\phi _{1}{\displaystyle \prod _{\ell \le m}}\left( 1-\overline{q}_{i_{\ell }}^{\textrm{S}}X_{i_{\ell }}\right) \right) $$

and the expected “disruption risk” cost of facility location:

$${\displaystyle \sum _{j\in J}}h_{j}\left( {\displaystyle \sum _{t\le n}}d_{jj_{t}}r_{jj_{t}}\prod _{\ell <t}\left( 1-\overline{q}_{j_{\ell }}^{\textrm{F}}Y_{j_{\ell }}\right) \overline{q}_{j_{t}}^{\textrm{F}}Y_{j_{t}}+\phi _{2}{\displaystyle \prod _{\ell \le n}}\left( 1-\overline{q}_{j_{\ell }}^{\textrm{F}}Y_{j_{\ell }}\right) \right) $$.

For each field *i*, the product $\prod _{\ell <s}\left( 1-\overline{q}_{i_{\ell }}^{\textrm{S}}X_{i_{\ell }}\right)$ determines the probability that the amount of agricultural waste at field *i* cannot be collected from any of its s-1 closest storage sites. Each expression $1-\overline{q}_{i_{\ell }}^{\textrm{S}}X_{i_{\ell }},\ \ell \le s-1$ in this product equates to 1 if a storage is not located at site $i_{\ell }$ (i.e., $X_{i_{\ell }}=0$) or $q_{i_{\ell }}^{\textrm{S}}$ if a storage is located at site $i_{\ell }$ (i.e., $X_{i_{\ell }}=1$). Therefore, to be collected from a storage located at the *s*th closest location (i.e., $X_{i_{s}}=1$), all storages located closer than position $i_{s}$ (i.e., at sites $i_{\ell },\ell \le s-1$) must fail, while the storage at $i_{s}$ must not fail (which happens with a probability $\overline{q}_{i_{s}}^{\textrm{S}}$). Hence, the combined term $\prod _{\ell <s}\left( 1-\overline{q}_{i_{\ell }}^{\textrm{S}}X_{i_{\ell }}\right) \overline{q}_{i_{s}}^{\textrm{S}}X_{i_{s}}$ gives the correct probability that field *i* will be collected from its *s*th closest site. The product ${\displaystyle \prod _{\ell \le m}}\left( 1-\overline{q}_{i_{\ell }}^{\textrm{S}}X_{i_{\ell }}\right)$ determines the probability that all *m* storages fail.

Similarly, for each storage *j*, the product $\prod _{\ell <t}\left( 1-\overline{q}_{j_{\ell }}^{\textrm{F}}Y_{j_{\ell }}\right)$ determines the probability that the amount of agricultural waste at storage *j* cannot be collected from any of its t-1 closest facility sites. Each expression $1-\overline{q}_{j_{\ell }}^{\textrm{F}}Y_{j_{\ell }},\ \ell \le t-1$ in this product equates to 1 if a facility is not located at site $j_{\ell }$ (i.e., $Y_{j_{\ell }}=0$) or $q_{j_{\ell }}^{\textrm{F}}$ if a facility is located at site $j_{\ell }$ (i.e., $Y_{j_{\ell }}=1$). Therefore, to be collected from a facility located at the *t*th closest location (i.e., $Y_{j_{t}}=1$), all facilities located closer than position $j_{t}$ (i.e., at sites $j_{\ell },\ell \le t-1$) must fail, while the facility at $j_{t}$ must not fail (which happens with a probability $\overline{q}_{j_{t}}^{\textrm{F}}$). Hence, the combined term $\prod _{\ell <t}\left( 1-\overline{q}_{j_{\ell }}^{\textrm{F}}Y_{j_{\ell }}\right) \overline{q}_{j_{t}}^{\textrm{F}}Y_{j_{t}}$ gives the correct probability that storage *j* will be collected from its *t*th closest site. The product ${\displaystyle \prod _{\ell \le n}}\left( 1-\overline{q}_{j_{\ell }}^{\textrm{F}}Y_{j_{\ell }}\right)$ determines the probability that all *n* facilities fail.

Constraints (2) are used to compute the expected amount of agricultural waste that a storage collects from fields. Similarly, as explained above, for each storage *j*, the product $\prod _{\ell <\overline{i}_{j}}\left( 1-\overline{q}_{i_{\ell }}^{\textrm{S}}X_{i_{\ell }}\right)$ determines the probability that the amount of agricultural waste at field *i* cannot be collected from any of its $\overline{i}_{j}-1$ closest storage sites. Hence, the combined term $\prod _{\ell <\overline{i}_{j}}\left( 1-\overline{q}_{i_{\ell }}^{\textrm{S}}X_{i_{\ell }}\right) \overline{q}_{j}^{\textrm{S}}X_{j}$ gives the correct probability that filed *i* will be collected from storage *j*. Then, the overall combined term $\sum _{i\in I}h_{i}\left( \prod _{\ell <\overline{i}_{j}}\left( 1-\overline{q}_{i_{\ell }}^{\textrm{S}}X_{i_{\ell }}\right) \overline{q}_{j}^{\textrm{S}}X_{j}\right)$ in the right hand side of (2) determines the expected amount of agricultural waste collected from all fields by storage *j*.

**Closest-rank mapping and induced probabilities.** For each field i∈I, we construct an ordered list of all candidate storage sites *J* by non-decreasing distance $d_{ij}$. Let $i_{s}$ denote the storage site occupying position s=1,…,m in this list (i.e., the *s*th closest storage to field *i*, in Table 2). For any storage j∈J, we define its rank relative to field *i* as $\bar{i}_{j}=s$, such that $j=i_{s}$. This ranking is fixed throughout the model and is used to compute compound probabilities:

- The probability that field *i* is served/ collected from storage site *j* equals

$$\prod _{\ell <\overline{i}_{j}}\left( 1-\overline{q}_{i_{\ell }}^{\textrm{S}}X_{i_{\ell }}\right) \overline{q}_{j}^{\textrm{S}}X_{j},$$

i.e., all sites closer than site *j* fail (or are not opened), and site *j* is opened.

- Consequently, constraints (2) compute the expected flow into storage *j* as

$$h_{j}=\sum _{i\in I}h_{i}\bigg (\prod _{\ell <\overline{i}_{j}}\left( 1-\overline{q}_{i_{\ell }}^{\textrm{S}}X_{i_{\ell }}\right) \overline{q}_{j}^{\textrm{S}}X_{j}\bigg ),$$

which is the sum, over all fields *i*, of demand $h_{i}$ weighted by the probability that field *i* is ultimately assigned to storage site *j*. This same ranking and probability logic appears in the field-storage component of the objective function (2) and, analogously, in the storage-facility component with $j_{t}$ for facilities.

Constraints (3) - (4) are that the exact number of opened storages and facilities, respectively. Constraints (5) - (6) are binary decision variables.

There exist nonlinear terms in the objective function (1) and constraints (2). This is a MINLP model that cannot be easily solved by any optimisation solver (e.g., CPLEX). We thus develop a two-stage “probability chains” technique to transform the MINLP model into a quadratic programming form that can be solved by an advanced optimisation solver, and propose a two-stage solution method to seek quickly the optimal solutions for the medium-sized instances.

### Two-stage “probability chains” technique

In this section, we develop a two-stage “probability chains” technique to solve simultaneously the reliable storage and facility location problems. To linearise the nonlinear terms of compound probabilities in the MINLP, we introduce the auxiliary variables for the following fields and storages:

**Figure:**
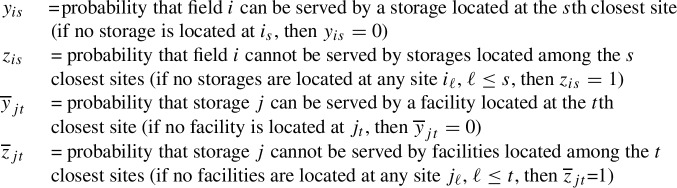

**Fig. 5:**
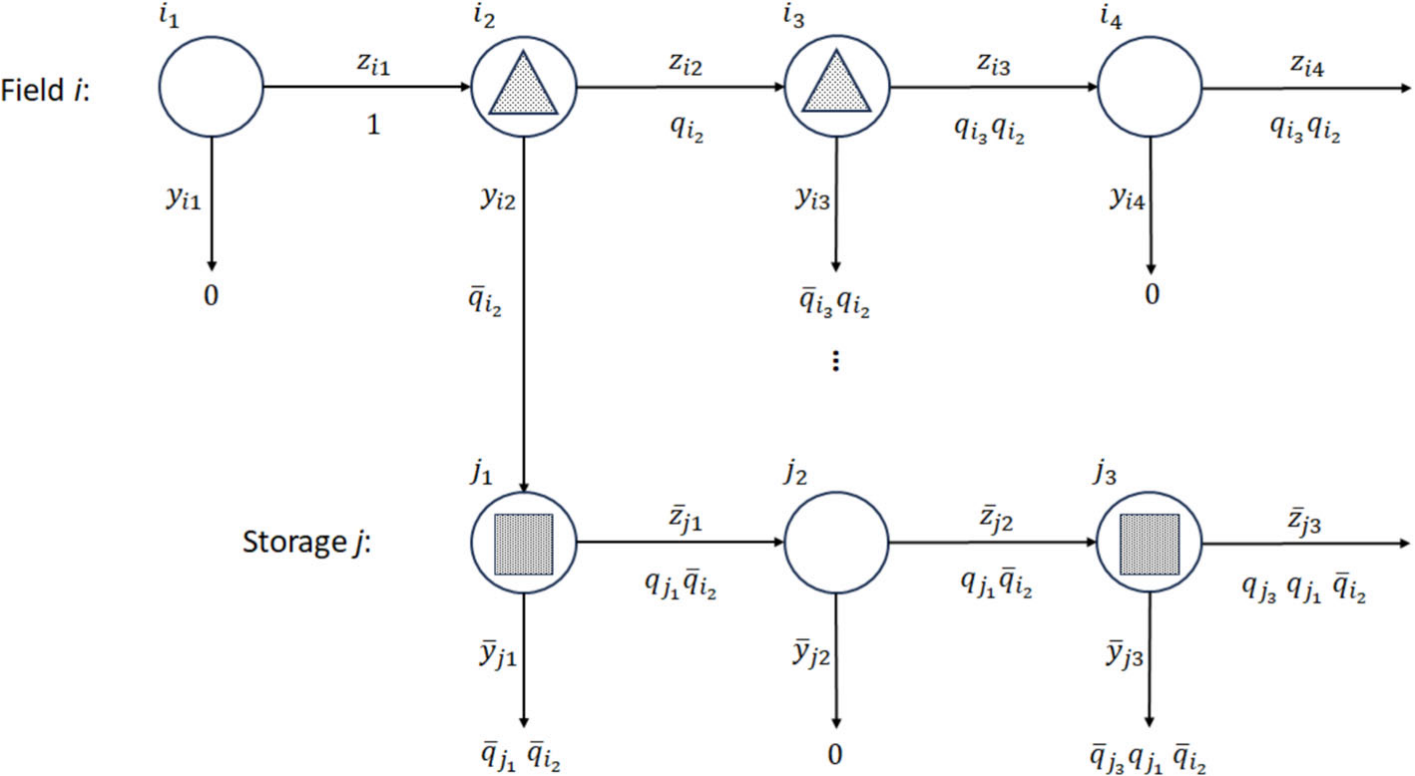
A graph representation of a hypothetical two-stage probability chain for a hypothetical field *i* that can be served (i.e., collecting agricultural waste) by m=4 sites (nodes) with $p_{1}=2$ storages (i.e., representing by dotted triangles) located at site $i_{2}$ and $i_{3}$, and a hypothetical storage *j* that can be served (i.e., collecting agricultural waste) by n=3 sites (nodes) with $p_{2}=2$ facilities (i.e., representing by dotted squares) located at site $j_{1}$ and $j_{3}$.

We design a hypothetical two-stage probability chain as shown in Figure 5. In this figure, stage 1 (for field *i*) is as same as the probability chain of (O’Hanley et al., 2013), while stage 2 (for storage *j*) is modified to compute probabilities that storage *j* can be served by a facility located at the *t*th closest site (as well as probabilities that storage *j* cannot be served by facilities located among the *t* closest sites). Stage-2 probabilities depend on stage-1 probabilities that field *i* can be served by a storage located at the *s*th closest site. For example, in Figure 5 stage-2 probabilities ($\bar{y}_{j1}$ and $\bar{z}_{j1}$) are affected by probability $(y_{i2})$ that field *i* can be served by a storage located at the 2nd closest site.

Next, a proposition is proposed to state that the two-stage probability chains follow from the law of total probability under independent failures, explaining that stage-2 chains are conditional on stage-1 activation.

**Proposition (two-stage probability chains):**

Assume independent site failures across storage and facility candidates. For each field *i*, let ($i_{1},\ldots ,i_{m}$) list storage candidates in non-decreasing distance $d_{ij}$; for each storage *j*, let ($j_{1},\ldots ,j_{n}$) list facility candidates in non-decreasing distance $d_{jk}$. Then,

- Stage 1 (field→ storage). The probability ($y_{is}$) that field *i* is ultimately served by storage $i_{s}$, and the probability ($z_{is}$) that field *i* cannot be served by any storage located among the *s* closest sites, respectively, equal

$$y_{is}=\left( \prod _{\ell <s}\left( 1-\bar{q}_{i_{\ell }}^{S}X_{i_{\ell }}\right) \right) \bar{q}_{i_{s}}^{S}X_{i_{s}},\mathrm {{\textstyle and}}\;z_{is}=\prod _{\ell \le s}\left( 1-\bar{q}_{i_{\ell }}^{S}X_{i_{\ell }}\right) .$$

This is the standard single-stage probability chain identity.

- Stage 2 (storage→ facility), conditional on stage-1 activation. Conditional on storage *j* actually receiving flow from stage 1, the probability ($\bar{y}_{jt}$) that storage *j* is ultimately served by facility $j_{t}$, and the probability ($\bar{z}_{jt}$) that storage *j* cannot be served by any facility located among the *t* closest sites, respectively, equal

$$\bar{y}_{jt}=\bigg (\prod _{\ell <t}\left( 1-\bar{q}_{j_{\ell }}^{F}Y_{j_{\ell }}\right) \bigg )\bar{q}_{j_{t}}^{F}Y_{j_{t}},\mathrm {{\textstyle and}} \,\, \bar{z}_{jt}=\prod _{\ell \le t}\left( 1-\bar{q}_{j_{\ell }}^{F}Y_{j_{\ell }}\right) .$$

Unconditioning over the stage-1 activation event yields the composite probabilities used in the objective function. Constraints (17) are precisely the activation constraints that ties stage 2’s base mass ($\bar{y}_{j1}+\bar{z}_{j1}$ ) to stage 1’s event probability $y_{is}X_{is}$ when $X_{is}=1$.

#### Proof

For stage 1, independence across storage sites implies

$$\text {Probability}\left\{ i\text { is served by }i_{s}\right\} =\text {Probability}\left\{ \bigcap _{\ell<s}\text {Event}_{i_{\ell }}^{fail}\right\} \times \text {Probability}\left\{ \text {Event}_{i_{s}}^{success}\right\} =\prod _{\ell <s}\left( 1-\bar{q}_{i_{\ell }}^{S}X_{i_{\ell }}\right) \bar{q}_{i_{\ell }}^{S}X_{i_{\ell }},$$

which defines $y_{is}$; the complementary event up to rank *s* gives $z_{is}$. For stage 2, it is conditional on the event that storage *j* is active, i.e., it receives flow from some field (law of total probability with respect to the sigma algebra generated by the stage-1 assignments). Given stage-1 activation, independence across facilities yields the same chain form for $\bar{y}_{jt}$ and $\bar{z}_{jt}$. Constraints (17) assign the total probability mass entering stage 2 to equal the activation probability from stage 1, thereby ensuring that stage-2 probabilities are conditional on stage-1 service occurrence and that these two chains compose without double counting. This is the principled two-stage analogue of the single-stage probability chains of O’Hanley et al. (2013) and our earlier usage Tran et al. (2017), generalized to include both node and link failures. □

The reduced non-linear version of RTLP-NP is then given by:

[RTLP-NP-reduced]:

7 $$\begin{aligned} &  \min \quad {\displaystyle \sum _{i\in I}}h_{i}\left( {\displaystyle \sum _{s\le m}}d_{ii_{s}}r_{ii_{s}}y_{is}+\phi _{1}z_{im}\right) +{\displaystyle \sum _{j\in J}}h_{j}\left( {\displaystyle \sum _{t\le n}}d_{jj_{t}}r_{jj_{t}}\overline{y}_{jt}+\phi _{2}\overline{z}_{jn}\right) \end{aligned}$$

8 $$\begin{aligned} &  \text {s.t.:}\ h_{j}=\sum _{i\in I}h_{i}y_{i\overline{i}_{j}},\ \forall j\in J, \end{aligned}$$

9 $$\begin{aligned} &  \quad \sum _{j\in J}X_{j}=p_{1}, \end{aligned}$$

10 $$\begin{aligned} &  \quad \sum _{k\in K}Y_{k}=p_{2}, \end{aligned}$$

11 $$\begin{aligned} &  \quad X_{j},Y_{k}\in \{0,1\},\ \forall j\in J,k\in K, \end{aligned}$$

12 $$\begin{aligned} &  \quad y_{i1}+z_{i1}=1,\ \forall i\in I, \end{aligned}$$

13 $$\begin{aligned} &  \quad y_{is}+z_{is}=z_{i(s-1)},\ \forall i\in I,\ s=2,...,m, \end{aligned}$$

14 $$\begin{aligned} &  \quad y_{is}\le \overline{q}_{i_{s}}X_{i_{s}},\ \forall i\in I,\ s=1,...,m, \end{aligned}$$

15 $$\begin{aligned} &  \quad y_{is}\le \overline{q}_{i_{s}}z_{i(s-1)},\ \forall i\in I,\ s=2,...,m, \end{aligned}$$

16 $$\begin{aligned} &  \quad y_{is},z_{is}\ge 0,\ \forall i\in I,\ s=1,...,m, \end{aligned}$$

17 $$\begin{aligned} &  \quad \overline{y}_{j1}+\overline{z}_{j1}=y_{is}X_{i_{s}},\ \forall i\in I,\ s=1,...,m,\ \forall j\in J, \end{aligned}$$

18 $$\begin{aligned} &  \quad \overline{y}_{jt}+\overline{z}_{jt}=\overline{z}_{j(t-1)},\ \forall j\in J,\ t=2,...,n, \end{aligned}$$

19 $$\begin{aligned} &  \quad \overline{y}_{jt}\le \overline{q}_{j_{t}}Y_{j_{t}},\ \forall j\in J,\ t=1,...,n, \end{aligned}$$

20 $$\begin{aligned} &  \quad \overline{y}_{jt}\le \overline{q}_{j_{t}}\overline{z}_{j(t-1)},\ \forall j\in J,\ t=2,...,n, \end{aligned}$$

21 $$\begin{aligned} &  \quad \overline{y}_{jt},\overline{z}_{jt}\ge 0,\ \forall j\in J,\ t=1,...,n. \end{aligned}$$

This is still a MINLP due to the remaining non-linear terms in (7) - (8) and (17). To linearise the non-linear terms, we apply the piece-wise linear approximation technique for the product of continuous variables on (7) - (8), and the linearization technique for the product of binary variable and continuous variable on (17).

Let $u_{ijt}=\frac{1}{2}\left( y_{i\overline{i}_{j}}+\overline{y}_{jt}\right)$ and $v_{ijt}=\frac{1}{2}\left( y_{i\overline{i}_{j}}-\overline{y}_{jt}\right)$ , $\overline{u}_{ij}=\frac{1}{2}\left( y_{i\overline{i}_{j}}+\overline{z}_{jn}\right)$ and $\overline{v}_{ij}=\frac{1}{2}\left( y_{i\overline{i}_{j}}-\overline{z}_{jn}\right)$, where $u_{ijt}\ge 0,v_{ijt}\in [-\frac{1}{2},\frac{1}{2}]\ \forall i,j,t$ and $\overline{u}_{ij}\ge 0,\overline{v}_{ij}\in [-\frac{1}{2},\frac{1}{2}]\ \forall i,j$. The objective function (7) and constraints (8) can be written as:

22 $$\begin{aligned} {\displaystyle \sum _{j\in J}}{\displaystyle \sum _{i\in I}\sum _{t\le n}h_{i}\left[ d_{jj_{t}}r_{jj_{t}}\left( u_{ijt}^{2}-v_{ijt}^{2}\right) +\phi _{2}\left( \overline{u}_{ij}^{2}-\overline{v}_{ij}^{2}\right) \right] }, \end{aligned}$$

that is a quadratic function.

Next, let $w_{is}=y_{is}X_{i_{s}}$, then $w_{is}\le X_{i_{s}}\boldsymbol{\textrm{M},}\ w_{is}\ge -X_{i_{s}}\boldsymbol{\textrm{M},}\ w_{is}\le y_{is}+\left( 1-X_{i_{s}}\right) \boldsymbol{\textrm{M},}\ w_{is}\ge y_{is}-\left( 1-X_{i_{s}}\right) \boldsymbol{\textrm{M}}$ with $\boldsymbol{\textrm{M}}$ is a big positive number, constraints (17) can be written as:

23 $$\begin{aligned} \overline{y}_{j1}+\overline{z}_{j1}=w_{is},\ \forall i\in I,\ s=1,...,m:X_{i_{s}}=1,\ \forall j\in J. \end{aligned}$$

The linearisations are algebraically exact, not ad hoc, because they rely on standard reformulations of bilinear probability expressions and binary–continuous products that preserve equivalence with the original nonlinear terms, rather than introducing heuristic approximations.

- The non-linearity arises from products of probabilities (continuous variables) and open/close decisions (binary variables), e.g., $y_{is}X_{i_{s}}$ in constraints (17), can be linearised. By introducing the midpoint reparameterization, we convert the continuous-and-continuous quadratic expressions to a mixed-integer quadratic programming in constraints (22). These are identity transformations (no approximation).
- We linearise binary-and-continuous products via a standard big **M** method to create $w_{is}=y_{is}X_{i_{s}}$in constraints (23), which enforces $w_{is}=0$ when $X_{i_{s}}=0$ and $w_{is}=y_{is}$ when $X_{i_{s}}=1$. This is also algebraically equivalent to the original bi-linear term.

Hence, the reformulated model is mathematically equivalent to the original MINLP representation of the same reliability expressions (not an ad hoc surrogate). This is a mixed-integer quadratic programming (MIQP) model that could be solved by advanced optimisation solvers (e.g., CPLEX). The complexity of this model is , particularly including |J|+|K| binary variables, $|I|\times |J|+|J|\times |K|$ continuous variables, and  constraints.

### Two-stage solution method

Due to the property of problem decomposition, we develop an efficient two-stage solution method for solving the RTLP, in which the reliable storage location problem (Stage 1) is first solved to collect agricultural waste from fields to storages, and then the reliable facility location problem (Stage 2) is solved (with the storages opened from solving the problem at Stage 1) to collect the waste from storages to facilities. For each reliable location problem, we apply “probability chains” technique (O’Hanley et al., 2013) to find the optimal locations that minimise total expected “disruption risk” cost.

The Stage-1 and Stage-2 models for the corresponding reliable storage location and reliable facility location problems are presented as follows.

[Stage 1]:

24 $$\begin{aligned} &  \min \ {\displaystyle \sum _{i\in I}}h_{i}\left( {\displaystyle \sum _{s\le m}}d_{ii_{s}}r_{ii_{s}}\prod _{\ell <s}\left( 1-\overline{q}_{i_{\ell }}^{\textrm{S}}X_{i_{\ell }}\right) \overline{q}_{i_{s}}^{\textrm{S}}X_{i_{s}}+\phi _{1}{\displaystyle \prod _{\ell \le m}}\left( 1-\overline{q}_{i_{\ell }}^{\textrm{S}}X_{i_{\ell }}\right) \right) \end{aligned}$$

25 $$\begin{aligned} &  \text {s.t.:}\ \sum _{j\in J}X_{j}=p_{1}, \end{aligned}$$

26 $$\begin{aligned} &  X_{j}\in \{0,1\},\ \forall j\in J. \end{aligned}$$

[Stage 2]:

27 $$\begin{aligned} &  \min \ {\displaystyle \sum _{j\in J:X_{j}=1}}h_{j}\left( {\displaystyle \sum _{t\le n}}d_{jj_{t}}r_{jj_{t}}\prod _{\ell <t}\left( 1-\overline{q}_{j_{\ell }}^{\textrm{F}}Y_{j_{\ell }}\right) \overline{q}_{j_{t}}^{\textrm{F}}Y_{j_{t}}+\phi _{2}{\displaystyle \prod _{\ell \le n}}\left( 1-\overline{q}_{j_{\ell }}^{\textrm{F}}Y_{j_{\ell }}\right) \right) \end{aligned}$$

28 $$\begin{aligned} &  \text {s.t.:} h_{j}=\sum _{i\in I}h_{i}\left( \prod _{\ell \le \overline{i}_{j}}\left( 1-\overline{q}_{i_{\ell }}^{\textrm{S}}X_{i_{\ell }}\right) \overline{q}_{j}^{\textrm{S}}X_{j}\right) ,\ \forall j\in J:X_{j}=1, \end{aligned}$$

29 $$\begin{aligned} &  \sum _{k\in K}Y_{k}=p_{2}, \end{aligned}$$

30 $$\begin{aligned} &  Y_{k}\in \{0,1\},\ \forall k\in K. \end{aligned}$$

Using the same notations from Section 3.4 for applying the “probability chains” technique (O’Hanley et al., 2013) to linearise above-presented [Stage 1] and [Stage 2] models, these models can then be written in the following linear forms:

[MILP-Stage 1]:

31 $$\begin{aligned} &  \min {\displaystyle \sum _{i\in I}}h_{i}\left( {\displaystyle \sum _{s\le m}}d_{ii_{s}}r_{ii_{s}}y_{is}+\phi _{1}z_{im}\right) \end{aligned}$$

32 $$\begin{aligned} &  \text {s.t.: }\ \sum _{j\in J}X_{j}=p_{1}, \end{aligned}$$

33 $$\begin{aligned} &  X_{j}\in \{0,1\},\ \forall j\in J, \end{aligned}$$

34 $$\begin{aligned} &  y_{i1}+z_{i1}=1,\ \forall i\in I, \end{aligned}$$

35 $$\begin{aligned} &  y_{is}+z_{is}=z_{i(s-1)},\ \forall i\in I,\ s=2,...,m, \end{aligned}$$

36 $$\begin{aligned} &  y_{is}\le \overline{q}_{i_{s}}X_{i_{s}},\ \forall i\in I,\ s=1,...,m, \end{aligned}$$

37 $$\begin{aligned} &  y_{is}\le \overline{q}_{i_{s}}z_{i(s-1)},\ \forall i\in I,\ s=2,...,m, \end{aligned}$$

38 $$\begin{aligned} &  y_{is},z_{is}\ge 0,\ \forall i\in I,\ s=1,...,m. \end{aligned}$$

[MILP-Stage 2]:

39 $$\begin{aligned} &  \min {\displaystyle \sum _{j\in J:X_{j}=1}}h_{j}\left( {\displaystyle \sum _{t\le n}}d_{jj_{t}}r_{jj_{t}}\overline{y}_{jt}+\phi _{2}\overline{z}_{jn}\right) \end{aligned}$$

40 $$\begin{aligned} &  \text {s.t.:} h_{j}=\sum _{i\in I}h_{i}y_{i\overline{i}_{j}},\ \forall j\in J:X_{j}=1, \end{aligned}$$

41 $$\begin{aligned} &  \sum _{k\in K}Y_{k}=p_{2}, \end{aligned}$$

42 $$\begin{aligned} &  Y_{k}\in \{0,1\},\ \forall k\in K, \end{aligned}$$

43 $$\begin{aligned} &  \overline{y}_{j1}+\overline{z}_{j1}=1,\ \forall j\in J:X_{j}=1, \end{aligned}$$

44 $$\begin{aligned} &  \overline{y}_{jt}+\overline{z}_{jt}=\overline{z}_{j(t-1)},\ \forall j\in J:X_{j}=1,\ t=2,...,n, \end{aligned}$$

45 $$\begin{aligned} &  \overline{y}_{jt}\le \overline{q}_{j_{t}}Y_{j_{t}},\ \forall j\in J:X_{j}=1,\ t=1,...,n, \end{aligned}$$

46 $$\begin{aligned} &  \overline{y}_{jt}\le \overline{q}_{j_{t}}\overline{z}_{j(t-1)},\ \forall j\in J:X_{j}=1,\ t=2,...,n, \end{aligned}$$

47 $$\begin{aligned} &  \overline{y}_{jt},\overline{z}_{jt}\ge 0,\ \forall j\in J:X_{j}=1,\ t=1,...,n. \end{aligned}$$

**Fig. 6:**
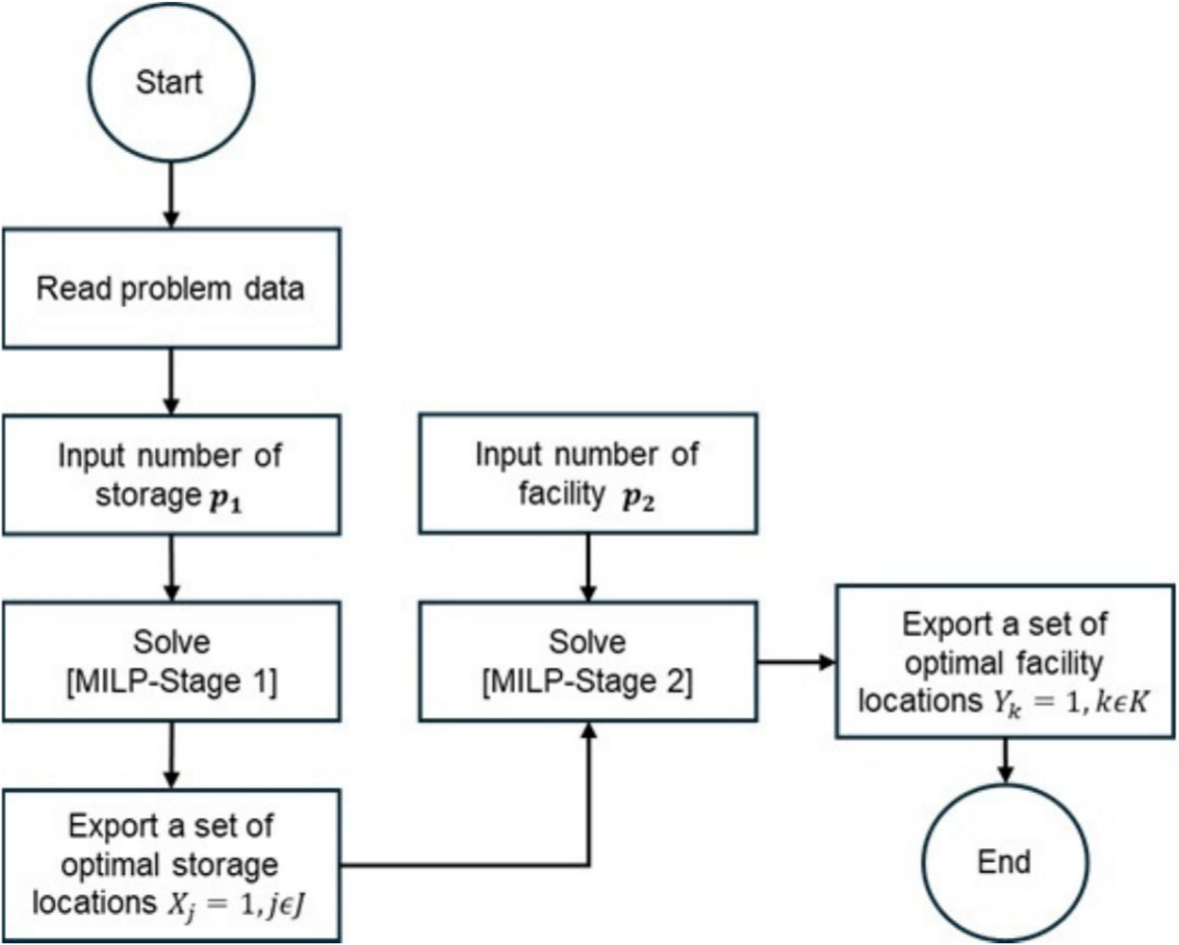
A flow chart of the two-stage solution method for RTLP.

An optimal solution for the RTLP is achieved by solving sequentially the [MILP-Stage 1] and [MILP-Stage 2] models. Figure 6 shows the flow chart of the two-stage solution method.

**Methodological note.** Although the proposed solution strategy is described as a “two-stage” solution method, the underlying model [RTLP-NP] is a single-level joint optimisation over storage and facility decisions. The sequential structure arises from the unidirectional dependence between stages: storage decisions determine field-and-storage assignments and induced flows $h_{j}$, while facility decisions do not influence the probability chains at storage stage. Solving the reliable storage location problem (Stage 1) first therefore does not restrict the feasible region or eliminate globally optimal (*X*, *Y*) combinations. Once *X* is fixed, the reliable facility location problem (Stage 2) can be solved exactly, yielding the best *Y* for that storage configuration.

### Math-heuristic algorithm

The two-stage solution method can seek the optimal solutions for the small-and-medium-sized instances. However, it could not achieve the optimal solutions for the large-sized instances within a reasonable computation time. It can be seen that the large number of fields mainly affect the computation time of solving the reliable storage location problem [MILP-Stage 1]. Hence, we develop an efficient math-heuristic algorithm, combing a meta-heuristic algorithm (e.g., the tabu search) to solve quickly and efficiently [MILP-Stage 1] and the mathematical programming model, i.e., solving [MILP-Stage 2] by an optimisation solver (e.g., CPLEX).

**Fig. 7:**
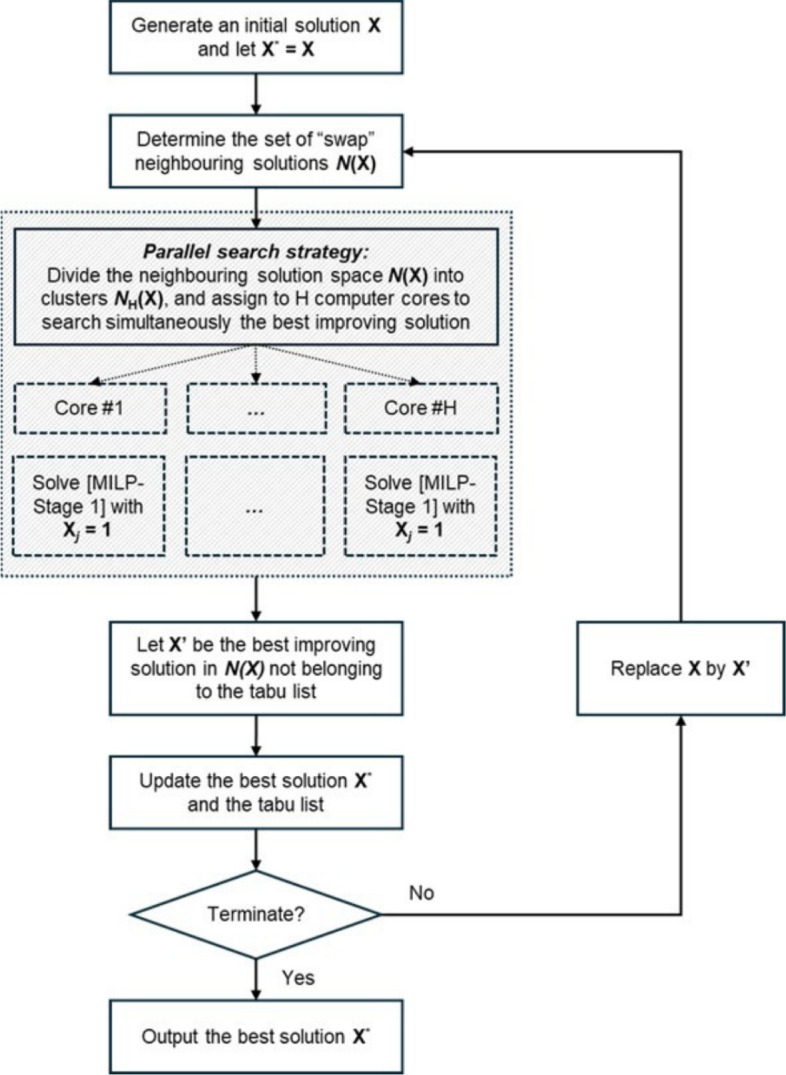
A flow chart of the tabu search for solving [MILP-Stage 1].

The overall flow chart of the math-heuristic algorithm is similar to that of the two-stage solution method (Figure 6) but solving [MILP-Stage 1] is replaced by the tabu search as shown in Figure 7. The main components of the tabu search are presented as follows.

#### Solution representation

**Fig. 8:**
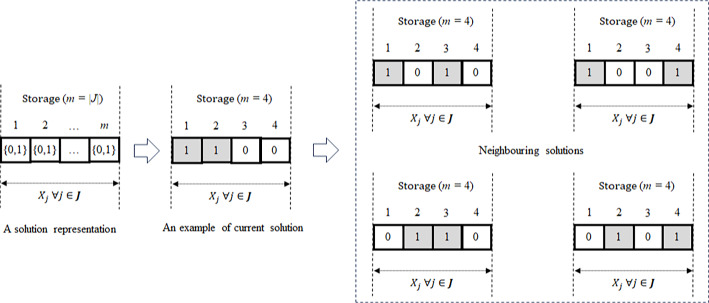
A solution representation, an illustration of the current solution and its neighbouring solutions.

A solution of [MILP-Stage 1] is represented by a list for storages consisting of *m* binary values $X_{j}\ \forall j\in J$ where $X_{j}=1$ if storage *j* is located. Figure 8 illustrates a solution representation for [MILP-Stage 1].

#### Initial solution

To construct an initial solution for the tabu search, we first determine for each storage site j∈J total expected “disruption risk” cost $\sum _{i\in I}h_{i}d_{ij}r_{ij}$ from all fields i∈I, and then sort the storage sites in ascending order of total expected “disruption risk” cost and select the first $p_{1}$ sites as storage locations.

#### Neighbourhood structure

A neighbourhood structure (namely, “swap”) is used to generate the set of neighbouring solutions for seeking the best improving solution. The “swap” neighbourhood structure is to exchange the status (i.e., open/close) between two storages. Figure 8 also illustrates the “swap” neighbourhood structure in an example of 4 storages. In the current solution, storages are located at sites 1 and 2. After applying the “swap” neighbouring structure, a set of 4 neighbouring solutions are achieved.

#### Tabu list

Tabu list prohibits recent combinations of moves from being chose in an attempt to prevent search from cycling back to previously visited solutions. Length of tabu list was determined based on preliminary setting.

#### Parallel search strategy

A parallel computing strategy is implemented for the tabu search. It aims to reduce the computation time involved with evaluating neighbouring solutions. By allocating a subset of neighbouring solutions to each central processing unit (CPU) core and then solving [MILP-Stage 1] model with the given values of $X_{j}=1,\ \forall j\in J$ for each neighbouring solution on different cores, we saved considerable computation time finding an improving tabu search move. Preliminary results showed that run times were roughly six times faster by using a parallel computing strategy.

#### Termination

Termination conditions for the tabu search include the maximum number of iterations or a time limit. If either condition is met, the tabu search terminates and the best solution found is reported.

## Numerical experiments

In this section, we investigate an efficacy of solving the RTLP by the proposed solution methods for sustainable agricultural waste collection, transport and treatment network design under impact of flooding in the Vietnam Mekong Delta. We evaluate the efficacy on two case studies in An Giang province, Vietnam (medium-sized instance) and the Vietnam Mekong Delta (large-sized instance). The solution methods were implemented in Visual Studio C++ and the mathematical programming models were solved by the IBM ILOG CPLEX version 12.9 callable library. The experiments were run on the HP Pavilion x360 Laptop with an Intel Core i7 1355U and 16 GB of RAM.

**Parameter tuning.** The parameters of tabu search were selected using a Design of Experiment (DoE) on a representative medium-sized instance (An Giang case study including 145 fields, 11 storages, and 7 facilities). In the DoE, iteration limits between 20-40 (with step = 5), tabu list lengths between 3-9 (with step = 2), and cluster counts between 2-8 (with step = 2) were tested. From the experimental results, we observed:

- **Maximum number of iterations:** During the experimental runs on the medium-sized instance, increasing the iteration limit beyond 30 produced no meaningful improvement in solution quality while increasing computation time almost linearly. The best improvement neighborhood moves typically stabilized between 20-25 iterations, so capping at 30 preserved solution quality while keeping computation time reasonable.
- **Tabu list length:** We tested the lengths of tabu list in the range 3-9. Lengths below 5 led to cycling (i.e., frequently revisiting recently explored storage configurations), whereas lengths above 7 slowed explorations without improving final solutions. A value of 5 made the best balance, avoiding cycling while keeping diversification broad enough to escape local minima.
- **Number of parallel clusters:** The parameter *H* = 8 matches the eight CPU cores (Intel Core i7 processor) used in all the computational experiments. Parallelising the neighbourhood evaluations yielded roughly a 6 times speed up. Fewer clusters increased computational time without improving solution quality.

Hence, the configuration (iterations = 30, tabu list = 5, and *H* = 8 parallel clusters), achieving the best objective value while using reasonable computation time on the 8-core computer, is used to solve the case studies.

### An example

We use an example of 5 fields, 3 storages and 2 facilities to demonstrate the optimal solution achieved by our solution method for the RTLP. Tables 3,4,5 show the data set of fields, storages and facilities, as well as the matrices of distance and disruption risk. The penalty cost for all storages failed (or all facilities failed) is 10,000. Number of opening storages is 2, and number of opening facilities is 1.

**Fig. 9:**
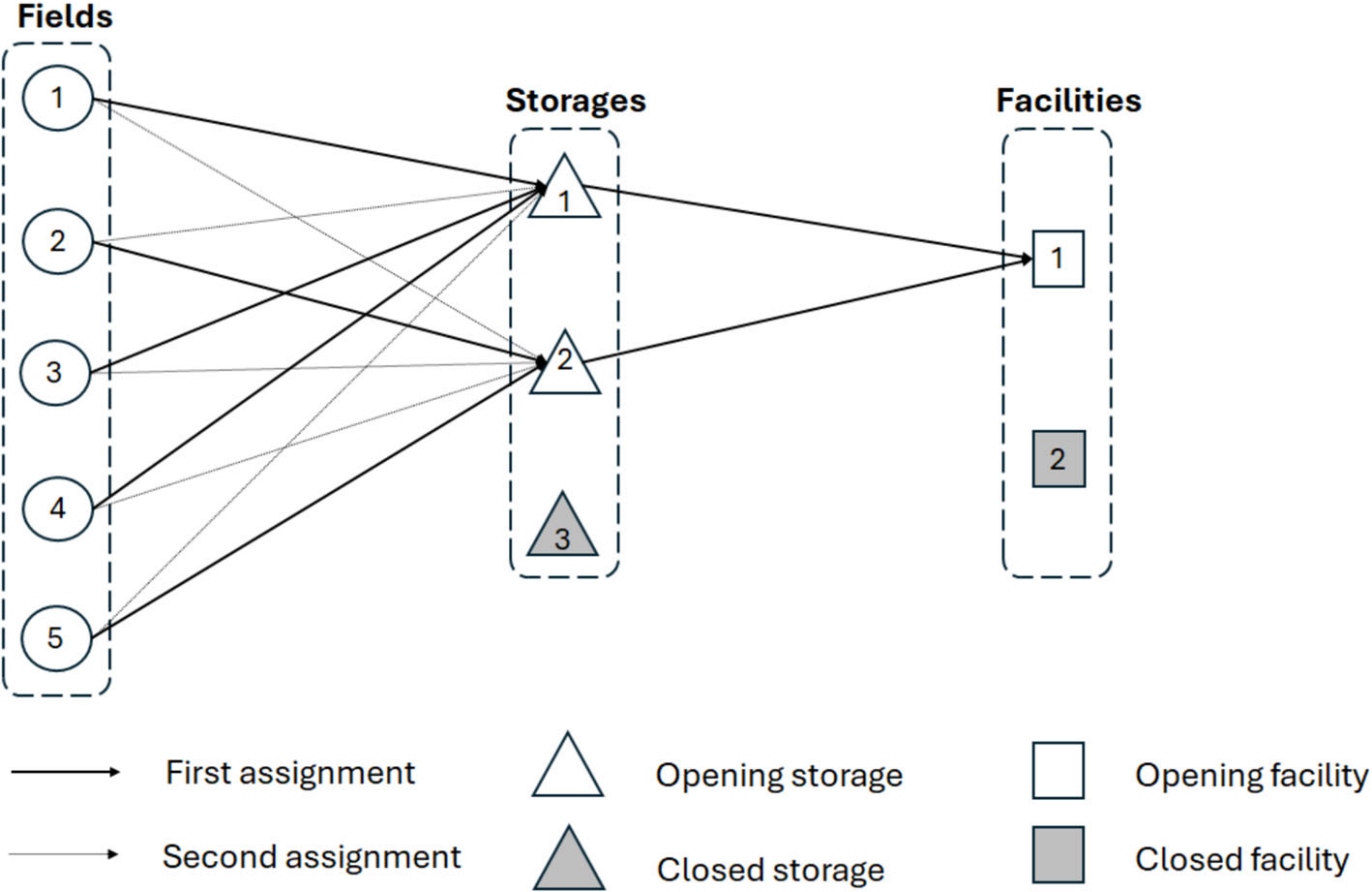
An illustration for the optimal assignment of fields, storages and facilities in the example.

The optimal solution by our model is shown in Table 6, in which two storages are opened at sites 1 and 2, and one facility is opened at site 1. As for the optimal assignment of fields and storages, the amount of agricultural waste at field 1 (C1) is collected by storage 1 (S1). If storage 1 (S1) fails, field 1 (C1) is served by storage 2 (S2). The amount of agricultural waste at field 2 (C2) is collected by storage 2 (S2). If storage 2 (S2) fails, field 2 (C2) is served by storage 1 (S1). A similar explanation is applied for the other fields. Because only one facility is opened, storages 1 and 2 (S1 and S2) are served by the facility (F1). Figure 9,10 demonstrates the optimal solution for this example.

**Table 3:** Fields.

Field	Demand (tonnes)		Storage	Risk		Facility	Risk
C1	5		S1	0.20		F1	0.10
C2	10		S2	0.30		F2	0.20
C3	5		S3	0.40			
C4	10						
C5	5

**Table 4:** Matrices of distance (km) and disruption risk for road network between fields and storages.

Field	Distance				Risk		
	S1	S2	S3		S1	S2	S3
C1	5	10	15		0.50	0.30	0.10
C2	10	5	15		0.30	0.50	0.10
C3	10	15	5		0.30	0.10	0.50
C4	5	10	15		0.50	0.30	0.10
C5	10	5	15		0.30	0.50	0.10

**Table 5:** Matrices of distance (km) and disruption risk for road network between storages and facilities.

Storage	Distance			Risk	
	F1	F2		F1	F2
S1	15	5		0.10	0.50
S2	5	15		0.10	0.50
S3	15	5		0.10	0.50

**Table 6:** The optimal assignment for the example.

Storage	Field						Facility	
	C1	C2	C3	C4	C5		**F1**	F2
**S1**	1	2	1	1	2		1	-
**S2**	2	1	2	2	1		1	-
S3	-	-	-	-	-		-	-

### Case studies

This section presents two case studies designed to evaluate the applicability, scalability, and performance of the proposed RTLP and solution methods under realistic conditions. The first case study focuses on An Giang province, representing a medium-sized, data-driven instance constructed from real geographic and flood-risk information. The second case study considers the broader Vietnam Mekong Delta, using a large-scale synthetic dataset to examine computational performance and solution robustness in a regional planning context. Together, these case studies illustrate how the proposed model supports reliability-oriented decision-making across different spatial scales.

#### Case study 1 (An Giang, Vietnam)

An Giang is known as one of the Vietnam Mekong Delta’s pioneer provinces in the development of biomass energy production from agricultural waste. Sustainable agricultural production and waste management models play a critical role to enhance the efficiency of biomass energy production network. The models ensure that agricultural waste from fields are fully collected, economically and transported to the reliable storages and treatment/ production facilities. Therefore, An Giang’s rural planners study innovative solutions to seek efficient and sustainable waste management models. The case study is constructed at An Giang province in the implementation of sustainable agricultural production and waste management model under impact of climate change (e.g., disruption risk by flooding).

In the case study, we consider 7 sites for locating facilities of biomass energy production and 11 storages to collect agricultural waste from 145 collection points (i.e., fields). Figure 5 illustrates the site candidates of 7 facilities (red squares), 11 storages (yellow triangles) and 145 fields (green circles). Distance matrices between fields and storages, and between storages and facilities are computed based on a real road network. Impact of flooding (e.g., disruption risk, measured by failure probability) on the sites of storages and facilities, and road network are determined based on the water level of areas from flood map generated by Vietnam Institute of Meteorology, Hydrology and Climate Change. Tables A1 - A6 (see Appendix) are the data of facilities, storages and fields in the case study. Due to the limitation of budget, An Giang Provincial Department of Agriculture and Rural Development would like to identify 8 optimal locations of storages and two optimal locations of facilities to minimise total expected “disruption risk” cost.

**Fig. 10:**
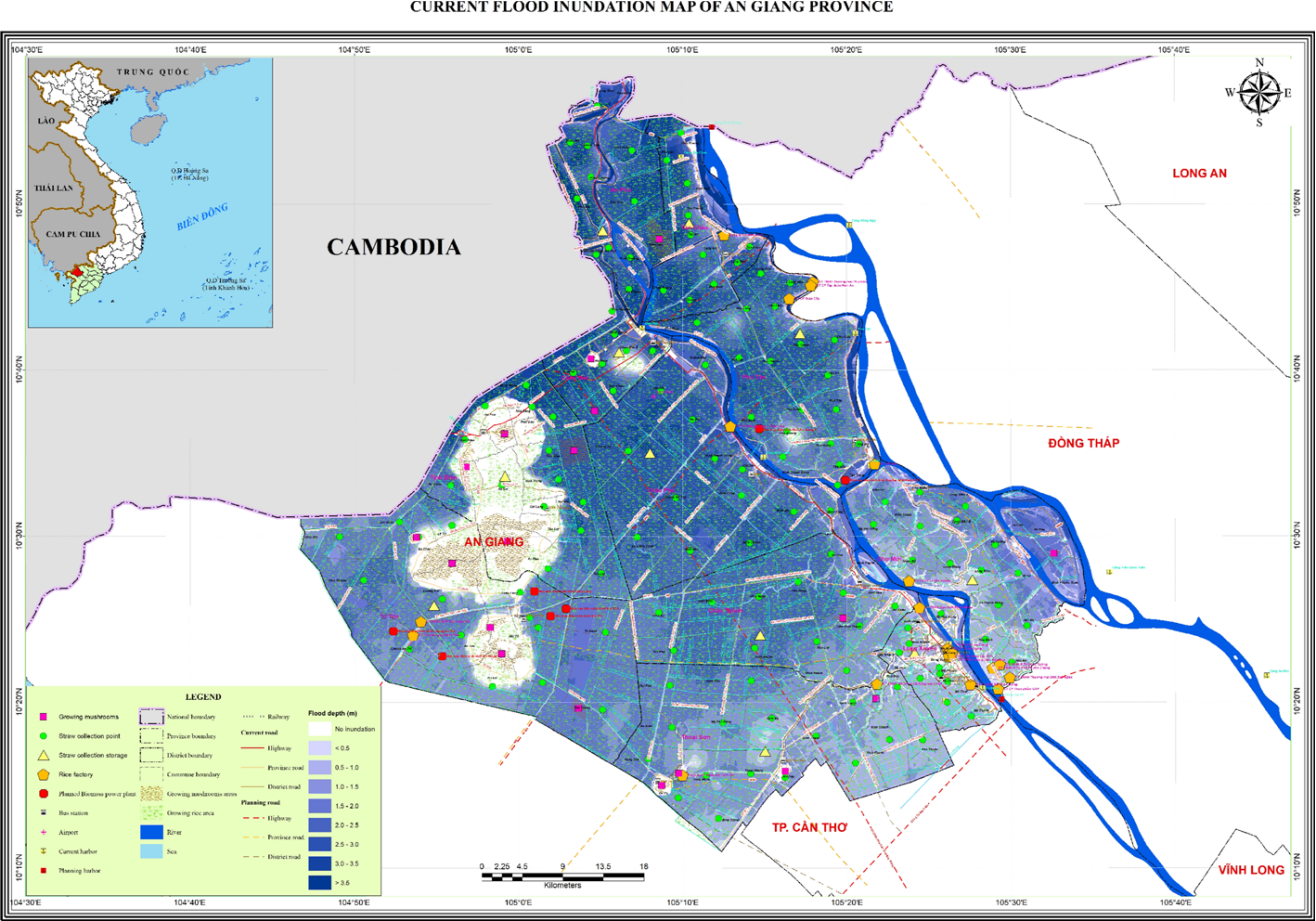
A map of site candidates for facilities and storages, and fields in An Giang, Vietnam.

#### Case study 2 (the Vietnam Mekong Delta)

**Fig. 11:**
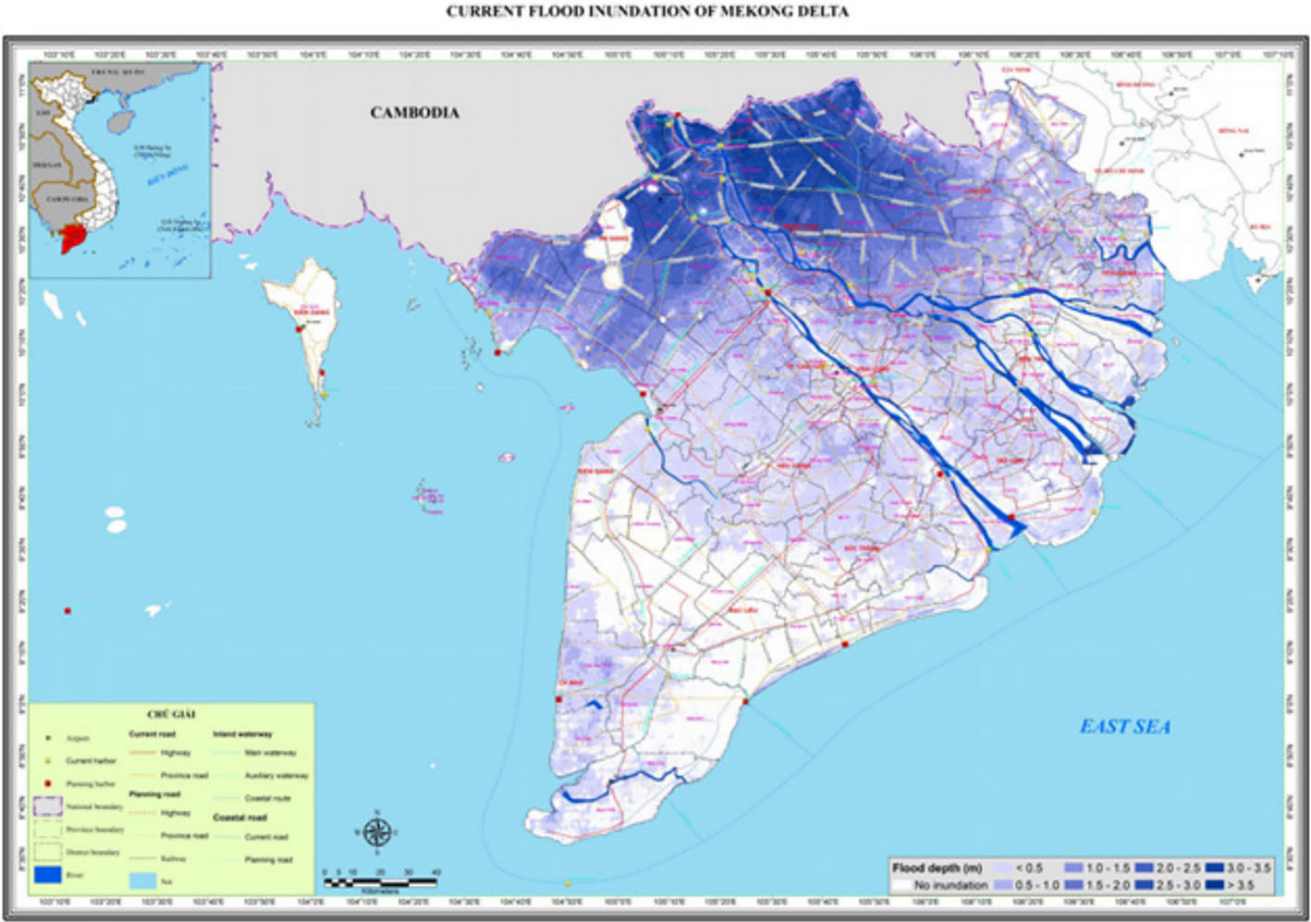
A predicted flooding map of the Vietnam Mekong Delta.

The Vietnam Mekong Delta consists of 13 provinces. Due to the issue of confidential data at some provinces, we use a synthesis data for the case study at the Vietnam Mekong Delta. In the case study, we consider 26 sites for locating facilities of biomass energy production (F1 - F26) and 143 storages (S1 - S143) to collect agricultural waste from 1,885 collection points (C1 - C1885). Demand of fields (tonnes) is a uniform distribution [1 - 10]. Distance matrix (km) between facilities and storages, as well as storages and fields are uniform distributions [10 - 100]. Again, the impact of flooding (i.e., disruption risk, measured by failure probability) on the sites of storages and facilities, and road network are determined based on the water level of areas from flood map generated by Vietnam Institute of Meteorology, Hydrology and Climate Change for the Vietnam Mekong Delta (see Figure 11). Due to the limitation of budget, Mekong River Commission Secretariat would like to identify 64 optimal locations of storages, and 14 optimal locations of facilities to minimise total expected “disruption risk” cost for the design of sustainable agricultural waste collection, transport and treatment network at the Vietnam Mekong Delta.

### Results and discussions

To compare the quality of solutions achieved by different solution methods, we compute the percentage deviation (denoted ▵) between the objective value found by a given solution method (denoted $XY_{Best}$) and the optimal value (denoted $XY^{*}$) or the lower bound (denoted LB) found by CPLEX. A computation time is given in CPU seconds.

$$\triangle ={\left\{ \begin{array}{ll} \begin{array}{cc} \frac{\left( {XY_{Best}}-{XY^{*}}\right) }{U^{*}}\times 100 & \text {if a known optimal solution exists},\\ \frac{\left( XY_{Best}-\text {LB}\right) }{\text {LB}}\times 100 & \text {otherwise}. \end{array}\end{array}\right. }$$

In addition to solving two case studies of An Giang and the Vietnam Mekong Delta (i.e., instances #1 and #5, respectively in Table 7), we construct other three instances (aka instances #2, #3 and #4) to evaluate the performance of our solution methods. The two-stage solution method is limited within 10 hours, while the math-heuristic algorithm terminates after 30 iterations.

Table 7 show the results for solving these instances. The two-stage solution method found the optimal solutions for the first three instances (medium-sized instances), but could not find the optimal solutions for the last two instances (large-sized instances) within 10 hours. For the largest-sized instance, it even is far away the optimal solution (i.e., a percentage deviation 90.54%). Math-heuristic algorithm (MHA) can achieve the optimal solutions for the first four instances, and a small percentage deviation (2.17%) for the last instance. The results show that the math-heuristic algorithm outperforms the two-stage solution method in terms of the quality of solution and computation time, especially for the large-sized instances. Therefore, the math-heuristic algorithm is applicable for solving the large-sized practical problems.

**Table 7:** The results of solving the case studies and instances.

Instances	Problem size						Two-stage solution method			MHA	
	|*I*|	|*J*|	|*K*|	$p_{1}$	$p_{2}$		▵	Time (s)		▵	Time (s)
1	145	11	7	8	2		0	0.88		0	0.75
2	290	22	10	16	3		0	4.78		0	4.14
3	580	33	14	24	5		0	198.35		0	63.72
4	1,160	66	20	32	7		5.66	36,000		0	615.28
5	1,885	143	26	64	14		90.54	36,000		2.17	2,030.54

As compared with the existing agricultural waste collection, transport and treatment networks under flooding impact, our innovative solution can reduce about 18% and 56% of total expected “disruption risk” cost for An Giang province and the Vietnam Mekong Delta, respectively. For the case study of An Giang province, the expected “disruption risk” cost reduction is because of

- Constructing the 8 storage sites outside high-risk flood zones (as determined by water-level maps).
- Relocating one facility site (e.g., existing site F2 to new site F7) away from flood-prone areas and rerouting flows through more reliable road segments.

### Comparison of the proposed RTLP vs. baseline models

Table 8 compares the proposed RTLP against two baseline models, such as a deterministic model (i.e., not considering any risk component) and a reliable single-stage facility location model (i.e., considering site failures only), on three instances (#1, #3, and #5 in Table 7). In particular, two baseline models are implemented to solve these instances under their own assumptions. Then, the optimal solutions (e.g., list of opened storages and/or facilities) obtained from the baseline models are used to compute total expected “disruption risk” cost and network reliability of these baseline models under the RTLP’s assumptions (i.e., considering both site and link failures). Next, we compute the percentage of relative expected cost increasing ($\triangle _{C}$) and relative reliability reduction ($\triangle _{R}$) by

$$\triangle _{C}=(C_{\text {M2 or M3}}-C_{\text {M1}})/C_{\text {M1}}\times 100$$,

$$\triangle _{R}=(R_{\text {M1}}-R_{\text {M2 or M3}})/R_{\text {M2 or M3}}\times 100$$,

where $C_{\text {M1}}$, $C_{\text {M2}}$ and $C_{\text {M3}}$ are total expected “disruption risk” cost, and $R_{\text {M1}}$, $R_{\text {M2}}$ and $R_{\text {M3}}$ are network reliability achieved by our RTLP model, the reliable single-stage facility location model, and the deterministic model, respectively.

The results demonstrate the “value of reliability” of our proposed RTLP. As compared with the deterministic model, the RTLP model achieves significantly lower in total expected “disruption risk” cost and higher in operational reliability due to its explicit treatment of both site and link failures at the beginning phase of network design. Relative to the reliable single-stage facility location model, the RTLP model also improves resilience by incorporating transport link disruptions and the two-stage (two-echelon) collection architecture. When increasing the size of instances, the baseline models increase more total expected “disruption risk” cost and reduce network reliability than our RTLP model. These comparisons highlight the long-term benefits of investing in reliability-oriented design of agricultural waste collection, transport and treatment network in flood-prone regions such as the Vietnam Mekong Delta.

**Table 8:** Comparative results of the proposed RTLP vs. the baseline models.

Instance	Model^*^	Risk components	Total expected “disruption risk”	Relative cost increasing	Network reliability	Relative reliability reduction
			cost (*C*)	(%)	(*R*, %)	(%)
1	M1	Site & link failures	4,782.90	-	95.94	-
	M2	Site failures only	5,548.80	13.80	94.25	1.79
	M3	None	5,662.09	15.53	90.18	3.39
3	M1	Site & link failures	10,278.28	-	99.96	-
	M2	Site failures only	13,838.36	25.73	96.82	3.24
	M3	None	14,647.17	29.93	92.67	7.87
5	M1	Site & link failures	23,988.44	-	~100.00	-
	M2	Site failures only	47,036.17	49.00	98.14	1.90
	M3	None	51,104.91	53.00	95.34	4.89

^*^Note that M1 = Our proposed RTLP, M2 = Reliable single-stage facility location model, and M3 = Deterministic model.

### Sensitivity analysis for penalty parameters

Because the numbers of storages and facilities are fixed, penalty parameters $\phi _{1}$ and $\phi _{2}$ only influence which sites are selected, not how many. Low penalties cause the model to prioritize shorter-distance locations (slightly higher risk), while high penalties shift selection toward lower-risk storages/ facilities (even if more distant). To evaluate the impact of the relative magnitude of $\phi _{1}$ and $\phi _{2}$ on the ranking of candidate sites, which can shift the solution toward more risk-resilient or more cost-efficient subsets, we do a parametric sensitivity analysis in which we vary $\phi _{1}$ and $\phi _{2}$ across a reasonable range.

In practice, the penalty values should be interpreted as order-of-magnitude representations of the economic and environmental consequences of complete service failure (e.g., emergency disposal, production interruption, or regulatory non-compliance), rather than calibrated monetary costs; the sensitivity analysis is designed to assess structural robustness of site selection under increasing disruption aversion.

Table 9 summarises the illustrative sensitivity of the optimal network design to variations in $\phi _{1}$ and $\phi _{2}$ for the An Giang case study. As the facility penalty increases, a clear change in the selected facility site is observed, with site F7 replacing F2 due to its lower failure probability. In contrast, the storage configuration remains unchanged across all scenarios, indicating robustness of the selected storage sites to penalty variation in this instance. Overall, the analysis suggests that increasing penalty parameters can progressively shift the model’s preference from cost-efficient locations toward more disruption-resilient sites, without altering the overall network size.

**Table 9:** Sensitivity analysis for penalty parameters on the An Giang case study.

Scenario			Value		Opening storages	Opening facilities	Total expected
$\phi _{1}$	$\phi _{2}$		$\phi _{1}$	$\phi _{2}$			“disruption risk” cost
Low	Low		1,000	1,000	1-3-5-6-7-8-9-11	1-2	1,886.01
Moderate	Moderate		5,000	5,000	1-3-5-6-7-8-9-11	1-2	3,368.75
High	High		10,000	10,000	1-3-5-6-7-8-9-11	1-7	4,782.90
High	Low		10,000	1,000	1-3-5-6-7-8-9-11	1-2	1,889.18
Low	High		1,000	10,000	1-3-5-6-7-8-9-11	1-7	4,786.07
Very high	Very high		100,000	100,000	1-3-5-6-7-8-9-11	1-7	28,589.46

## Conclusion of impact, limitation and future work

In this paper, a sustainable agricultural production and waste management model is proposed to stop burning directly agricultural waste at fields after each harvesting season, and to encourage utilising the agricultural waste to produce bio-organic fertilizer and biomass energy. As a result, it will improve the quality of environment and enhance the development of regional economy and society. Furthermore, the innovative practices aim towards four Sustainable Development Goals (SDGs) of United Nations, including SDG9 - Industry, Innovation and Infrastructure, SDG12 - Responsible consumption and production, SDG13 - Climate Action, and SDG17 - Partnerships for the goals.

A MINLP model is formulated to support rural planners to design the sustainable agricultural waste collection, transport and treatment network under impact of climate change (e.g., disruption risk due to flooding). In particular, the MINLP model minimises total expected “disruption risk” cost of locating storages and facilities, and collecting and transporting the agricultural waste from fields to storages, and from storages to treatment facilities. A two-stage “probability chains” technique is developed to transform the MINLP model into an MIQP model to seek the optimal solutions of the RTLP. A two-stage solution method, based on the property of problem decomposition, is then proposed to solve efficiently the problem. In addition, a math-heuristic algorithm, combing the tabu search and solving the MILP model, is constructed for solving the large-sized instances within a reasonable computation time.

The efficiency of the proposed solution methods is evaluated on the case studies in the Vietnam Mekong Delta, where the biomass energy production from agricultural waste is considered a high-priority task and a critical strategy towards sustainable agricultural production and waste management. As compared with the existing operational configuration in An Giang province, implementing the RTLP optimised network reduces total expected “disruption risk” cost by 18% and lowers the likelihood of systemic failure, while enabling more reliable straw collection that yields substantial environmental benefits. These results provide planners with a concrete, risk aware basis for selecting storage and facility locations, prioritizing investments outside flood-prone zones, and evaluating the long-term cost and emission savings achievable through reliable waste collection infrastructure. The results on the case studies show that our solution methods can achieve the optimal solutions for the large-sized sustainable agricultural waste collection, transport and treatment network design. Hence, they are capable to accelerate the world-wide impact of our innovative solutions of the sustainable agricultural production and waste management model.

**Impact:** This is an international research collaboration project, funded by UK Foreign, Commonwealth & Development Office, towards the sustainable agricultural production and waste management in ASEAN countries. In Phase 1 (2020/21), this project was implemented and brought the benefits of environment, economy and society for the farmers at Quang Tri, Central Vietnam, such as:

- **[Environment]** Mitigated burning 6.5 million tonnes (rice straw) directly on the fields, which reduced 5.6 million tons of CO2, 151 thousand tons of CO and 3.2 thousand tons of NOx into the atmosphere, and 9.7 tons of pesticide and fertilizer containers (toxic waste); Reduced using 2.1 million tons of traditional inorganic (harmful) fertilizer.
- **[Economy]** Contributed US$25 millions into Vietnam’s fertilizer market (US$264 millions, 2024).
- **[Society]** Reduced the number of people having respiratory issues and the number of accidents due to smoky environment from burning agricultural waste; Increased the household income about VND 10 millions (US$400) per crop season from selling rice straw to the bio-organic fertilizer production facility; Increased the employment rate in the province (i.e., thousands of jobs created by opening new production facilities); Increased the number of male workers back to rural areas to work on the bio-organic fertilizer production facilities, which improve gender imbalance issue at the province.

In Phase 2 (2023/24), we thus expect a significant long-term impact of environment, economy and society for the farmers at the Vietnam Mekong Delta, Southern Vietnam:

- **[Environment]** To mitigate burning 20.6 million tonnes (rice straw) directly on the fields; To reduce using 6.6 million tons of traditional inorganic (harmful) fertilizer.
- **[Economy]** To contribute US$80 millions into Vietnam’s fertilizer market.
- **[Society]** To reduce the number of people having respiratory issues and the number of accidents due to smoky environment; To increase the household income from selling rice straw to the biomass energy production facility; To increase the employment rate; To improve gender imbalance issue from increasing the number of male workers back to rural areas to work.

**Limitation:** In real-world settings, the selection of storage and facility sites is constrained by factors such as land tenure arrangements, competition from informal waste markets, administrative approval processes, need for economic or behavioral incentives for adoption. These are highly relevant considerations, particularly in agricultural waste systems where land ownership is fragmented and informal actors often dominate collection and trading activities. Hence, real-world implementation depends on:

- Land availability and tenure restrictions, which may prevent the development of storage or treatment sites at technically optimal locations;
- Informal market dynamics, where existing buyers or local waste handling practices can influence the feasibility of new facilities;
- Economic and policy incentives, which may be required for farmers and private operators to participate in a formalized collection network.

These factors do not alter the mathematical formulation of this optimisation model, but they are critical for practical deployment.

**Future work:** In practice, rice straw has a short post-harvesting collection window, and delays caused by flooding can significantly worsen collection feasibility and economic outcomes. Hence, incorporating time-sensitivity, such as dynamic flood progression, harvest schedules, or time dependent link availability, would further strengthen the problem’s realism and highlight the operational consequences of flood induced delays. A future research direction could be extending the model to capture both spatial reliability and time-sensitivity agricultural operations in an integrated way by multi-period or dynamic stochastic optimisation.

Another promising direction for future research is to extend the current static probability formulation into a multi-stage stochastic model capable of representing the temporal evolution of flooding conditions throughout the monsoon season. In practice, road accessibility and site reliability fluctuate as water levels shift across minor, moderate, and major flood stages, and incorporating these dynamics would enable the model to capture a richer set of operational realities. A time indexed or stage based formulation, where each flood state is associated with its own link and site failure probabilities, would allow rural planners to evaluate how infrastructure choices perform under varying hazard intensities, as well as identify critical periods when system vulnerability peaks. Moreover, the two-stage “probability chains” framework developed in this study can be naturally adapted to accommodate state dependent transitions and time varying activation events, making it well suited for dynamic extensions. Developing such a model would provide a more nuanced and seasonally responsive design tool, enhancing the practical relevance of reliability-based agricultural waste collection, transport, and treatment network design under climate driven flood variability.

Finally, a natural extension of the current framework is to relax the conditional independence assumption between link and site failures and explicitly model correlated flood induced disruptions. In many flood events, co-movement in failures arises when a regional surge simultaneously elevates the probability of road submergence and the failure of nearby storages or facilities. To capture this, three compatible pathways are proposed to preserve the “probability chains” structure and the decomposition logic developed: (i) a flood state mixture model, (ii) spatial zone latent factors, and (iii) a distributional robust surrogate. Each pathway nests the current model as a special case.
